# How Should We Treat Meningeal Melanocytoma? A Retrospective Analysis of Potential Treatment Strategies

**DOI:** 10.3390/cancers14235851

**Published:** 2022-11-27

**Authors:** Sarah Ricchizzi, Marco Gallus, Walter Stummer, Markus Holling

**Affiliations:** Department of Neurosurgery, University Hospital Muenster, 48149 Muenster, Germany

**Keywords:** meningeal melanocytoma, brain tumor, melanocytic tumors

## Abstract

**Simple Summary:**

As a rare tumor disease, only single case reports and small case series have been published on meningeal melanocytomas. In the case of complete surgical resection, there is a shallow risk of recurrence, whereas the benefit of radiotherapy or chemotherapy, both as single or combined therapy, is unclear. This work aims to analyze and summarize previously published cases and proposes a therapeutic algorithm.

**Abstract:**

Background: Meningeal melanocytomas (MM) are rare primary melanocytic tumors of the leptomeninges with an incidence of 1:10,000,000. Until now, there has been only sparse information about this tumor entity. Here, we provide a meta-analysis of all cases published in the English language since 1972. Methods: A literature review was performed using PubMed and Web of Science. All published cases were evaluated for location, sex, age, therapeutic approach, and outcome. In total, we included 201 patient cases in our meta–analysis. Results: The majority of MM was diagnosed more frequently in men between the third and fifth decade of life. Surgery is the preferred therapeutic approach, and total resection is associated with the best outcome. Patients with partial resection or tumor recurrence benefit from adjuvant radiotherapy, whereas chemo- or immunotherapies do not improve the disease course. Malignant transformation was described in 18 patients. Of these, 11 patients developed metastasis. Conclusions: We present the first retrospective meta-analysis of all MM cases published in the English language, including an evaluation of different treatment strategies allowing us to suggest a novel treatment guideline highlighting the importance of total resection for recurrence–free survival and characterizing those cases which benefit from adjuvant radiotherapy.

## 1. Introduction

Meningeal melanocytomas (MM) are rare primary melanocytic tumors of the leptomeninges. They can be divided into circumscribed or diffuse, benign or malignant lesions. Well–differentiated, circumscribed tumors are called meningeal melanocytomas, while their malignant counterparts are meningeal melanomas. Additionally, MM with increased mitotic activity or invasion of the CNS parenchyma are considered intermediate-grade lesions. Furthermore, diffuse melanocytic tumors are characterized by invasion into subarachnoid space. Depending on the dignity of the histological phenotype, the lesion is called meningeal melanocytosis or meningeal melanomatosis [1]. Since their first description in 1972 [2], about 201 cases have been reported in English worldwide. They can be found anywhere along the neuraxis, and clinical presentation depends on tumor location and size.

Depending on the amount of melanin [3], MM appear isointense to hyperintense on T1-weighted and isointense to hypointense on T2-weighted MRI and show heterogenous contrast enhancement. In CT, MM appear as well–defined, isodense to hyperdense with homogeneous, contrast-enhancing lesions [4,5].

**Figure 1:**
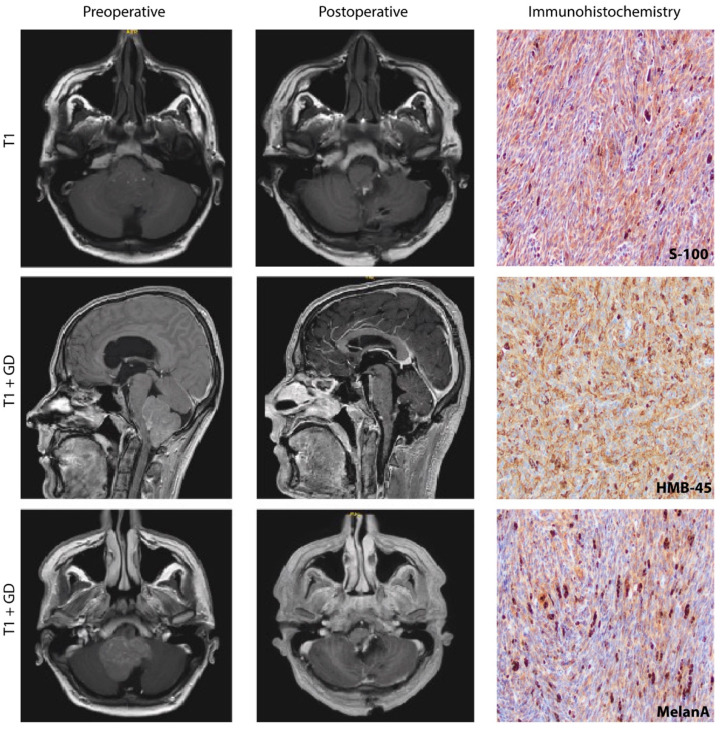
Exemplary perioperative MRI sequences of a 25-year-old Meningeal Melanocytoma (MM) patient that underwent surgery for a cerebellar lesion at our department. Axial T1 MRI showing a grey matter isointense posterior fossa mass shifting the brain stem forward, compressing cerebellum and brainstem, thereby leading to obstruction of the fourth ventricle. Preoperative (**left row**) T1 imaging after gadolinium application shows sagittal and axial inhomogeneous contrast enhancement. Postoperative (**middle row**) T1-weighted MRI without contrast agent (axial) and following gadolinium application (sagittal, axial), showing total resection of the mass with decompressed 4th ventricle and without secondary bleeding. Histopathological analysis of our case revealed spindle-shaped cells that showed expression of S-100, HMB45, and MelanA (**right row**).

Macroscopically the tumor is encapsulated without infiltration of the surrounding tissue [6,7]. The color of the tumor appears dark and varies between coal black, reddish brown, and dark blue [8]. Often, the dura appears darker than usual [9,10]. Microscopically, MM are characterized by nets composed of spindle-shaped cells [11] that express characteristically S–100, a typical calcium-binding protein [12,13], Melanoma Antigen (Melan–A), a melanocytic differentiation marker, and show positive reactivity for homatropine methyl bromide-45 (HMB–45) [10], a monoclonal antibody that interacts with GP-100. Furthermore, they show variable expression of neuron-specific enolase and vimentin [14] (Figure 1).

Although several case reports have been published, so far, it remains unclear which is the best treatment approach for MM, in particular for those cases in which only incomplete surgical resection could be performed. Therefore, the aim of this study was to reanalyze all published cases, as well as one unpublished case from our department, concerning the applied treatment strategies and their outcome.

## 2. Materials and Methods

### Search Strategy and Statistics

**Figure 2:**
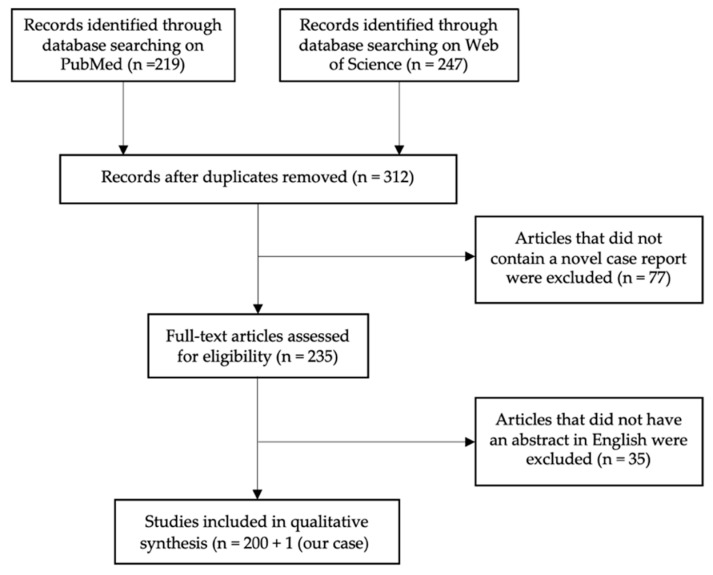
PRISMA Flow Chart visualizing the literature selection process. As database PubMed and Web of Science were used. As keyword “meningeal melanocytoma” was applied. In total, 219 results were found using PubMed and 247 using Web of Science published between the first description in 1972 and 2022. After subtracting duplicates, the total data covered 312 items. A total of n = 77 were excluded because no novel case report of MM was provided, and n = 35 were excluded as no abstract was available in English language. Therefore, in total, 201 published cases, as well as 1 unpublished case from our department, were included for further analysis.

MM cases were identified by using PubMed (Medline) and Web of Science (Clarivate). As the keyword, “meningeal melanocytoma” was used. In total, 219 (PubMed) and 247 (Web of Science) results were found, which were published between 1972 and 2022. After subtracting duplicates, the total data covered 312 items. Articles were excluded if they did not provide a novel case report, if the abstract did not include MM as a topic (n = 77), or if written in another language than English (n = 35), so in total, 201 cases were included in this analysis, as we also included one unpublished MM case from our department (Figure 1). All published cases were evaluated for age, gender, tumor location, and therapeutic approach as well as postoperative outcome (recurrence, metastasis, recurrence-free survival) (Figure 2).

All patient cases were included in our analysis, of which at least one of the above variables could be collected. For this reason, the population groups for the different analyses also differed in size (indicated each time in the text). Histological diagnoses of tumors described here were adopted from the original manuscripts. No reevaluation of histological specimens was performed by local neuropathologists according to the 2021 WHO classification. Therefore, the lesions depicted here were classified according to the current WHO classification in each case. Statistical analysis was performed using the statistics software SPSS (IBM, version 28). Descriptive data included the calculation of the mean or median and standard deviation. Pearson Chi–Square testing was performed to compare categorical variables. *p* values less than 0.05 were considered statistically significant.

## 3. Results

### 3.1. Population

#### 3.1.1. Age and Gender

**Figure 3:**
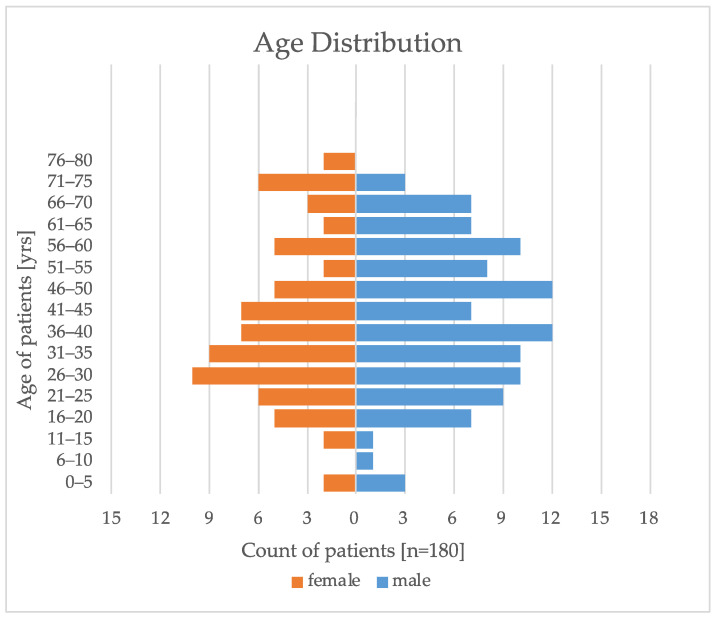
Age–dependent incidence of MM in females (red) and males (blue). The y–axis represents the age of the patients, and the x–axis is the count of female or male patients that were diagnosed with MM. The youngest patient was only 28 weeks old, and the oldest patient was diagnosed at the age of 79 years (Figure 2). The median age of disease onset of female patients was 37 years, and for male patients, it was 42 years. A higher frequency of MM was found in men compared to women (1.4:1).

Since 1972, 201 cases of MM were reported (Table S1). In 89.6% (180/201) of the reported cases, information concerning gender and age was provided. MM usually was diagnosed between the 3rd and 5th decade of life, with a median age of onset of the disease at 38 years. The youngest patient was only 28 weeks old, and the oldest patient was diagnosed at the age of 79 years (Figure 3). MM occurred more frequently in men (107/180, 59.4%) than in women (73/180, 40.6%). Females were diagnosed earlier (median, 37 years) than males (median, 42 years).

#### 3.1.2. Location

The location of the tumor was reported in 189 out of 201 cases. About half of the MM were found intracranially (101/189, 52.6%). The predominant location was the posterior fossa (57/101, 56.4%), followed by the middle cranial fossa with 11 cases (11/101, 10.9%) and the sellar region with 9 cases (9/101, 8.9%). Within the posterior fossa, MM occurred most often at the cerebellopontine–angle (CPA) (18/57, 31.6%). In the spine, MM occurred predominantly in the thoracic spine (39/78, 50%) and cervical spine (26/78, 33.3%). Furthermore, in eight cases, tumors grew in orbit. Two cases reported multifocal location within the spine as well as intracranially.

### 3.2. Treatment Strategies 

#### 3.2.1. Total Resection and Partial Resection

Treatment strategies were reported in 186 out of 201 cases (92.5%). As the primary therapeutic approach, surgery was performed in 179 out of 186 patients (96.2%) of the cases. In the remaining cases, radiotherapy was used in two, and no therapy was applied in five others. Total resection was achieved in 89 out of 179 (49.7%) of cases and partial resection in 73 out of 179 cases (40.7%), while in 9.5% of surgical procedures, the extent of resection was not documented (17/179).

#### 3.2.2. Adjuvant Therapy

Total resection was performed without any further therapy in 81 out of 179 patients (45.3%). The combination of total resection and radiotherapy was applied in 8 (4.5%), partial resection alone in 45 cases (25.1%), and adjuvant radiotherapy after partial resection in 24 out of 179 cases (13.4%). In two cases, radiosurgery was used after partial resection so that the remaining tumor could also be targeted [15,16]. Due to the severity and progression of the disease, five patients did not receive any therapy, and three of them died; one developed tumor progression, and for the last one, follow–up data were not available. In fact, one of those patients was diagnosed by autopsy.

Chemo- or immunotherapy such as Temozolomide [17], Cisplatin and Fotemustine [18,19], Methotrexate [20], Nivolumab [21], or Ipilimumab [22] were applied in 11 patients combined with radiotherapy (Table 1) [18,21,23]. However, in all patients but one, tumor growth was observed, and all patients but four died.

#### 3.2.3. Definite Radiotherapy and Radiosurgery

Due to tumor localization in an eloquent region, resection could not be performed in three patients. In two of these patients’ definite radiotherapy was chosen as a treatment option with a tumor-free follow-up of 42 months in 1 patient [24]. The other patient died due to pneumonia, unrelated to the MM [24]. In one case, radiosurgery alone was used as initial treatment after biopsy of the tumor [25].

**Table 1:** Overview of all cases in which adjuvant chemo- or immunotherapy was applied: In all cases, the adjuvant therapy failed, and tumor progression was observed. Ten patients died because of MM growth and its consequences.

Case	Age	Sex	Location	Treatment	Outcome
[17]	20	M	Intracranial	PR + RT, Reop + RT + Temolozomide + Cisplatin + Fotemustine	Death
[18]	46	F	Intracranial	TR, Reop + Fotemustine + Temolozomide	Death
[19]	38	M	Intracranial	TR, RT + Temolozomide	Death
[20]	79	F	Spine	PR, RT + Methotrexat	Death
[21]	70	M	Spine	PR + RT + Nivolumab, Reop + Temozolomide	Death
[22]	43	F	Intracranial	TR + RT, Reop + RT + Temozolomide + Ipilimumab	Death
[23]	71	F	Spine	TR + RT, PR + RT + C. parvum + Dactinomycin + Dacarbazine	Tumor progression
[25]	32	M	Orbita	Radiosurgery + Immunotherapy	Tumor progression
[26]	37	F	Intracranial	PR, Reop + RT + Temozolomide	Death
[27]	19	F	Spine	TR, PR + RT + Pembrolizumab + Bevacizumab + Temozolomide	Death
[28]	71	F	Spine	PR + RT, Reop + C. parvum, Dimethyl Triazeno Imidazole Carboxamide + Actinomycin	Death
[29]	36	F	Spine	TR, Reop + RT + Nivolumab	Death
[30]	35	M	Orbita	PR + RT + BCNU + DTIC + Cisplatin	Tumor progression
[31]	49	M	Orbita	PR + Dacarbazine + Vincristine + Nimustine Hydrochloride	No recurrence

Re–operation was the main therapy chosen in cases of tumor regrowth in 14 out of 44 cases (31.8%), followed by combination of re-operation and radiotherapy, applied in 7 (15.9%), re–operation combined with radiochemotherapy in another 4 (9.1%), while radiotherapy alone was used in 4 out of 44 cases (9.1%). In addition, three cases in which radiosurgery was used as follow-up therapy after recurrence of the tumor were also reported [32,33,34].

### 3.3. Outcome

For outcome analysis, we were able to evaluate 147 out of 201 data sets comprising information about initial therapy and follow-up (73.1%). Total resection was the most efficient therapy and showed a tumor-free interval without recurrence in 68.1% of cases (*p* = 0.001). The median tumor-free interval was 18 months, with a minimum of 1 month and a maximum of 35 years. If, alternatively, only partial resection was performed, a better outcome was shown in 61.9% of the patients by the additional use of adjuvant radiotherapy (see Table 2 and Table 3). Tumor progression was recorded here at a median of 24 months, ranging from 1 month to 16 years before recurrence developed.

When resection was combined with chemotherapy, tumor–free follow–up was not recorded in 10 out of 11 cases for total resection, partial resection, or with additional radiotherapy. Only one case was noted to have a tumor-free interval [31] (Table 1).

For radiosurgery, the outcome in all but one case was found to result in a tumor–free or progression–free interval. For this, it has been regardless of whether radiosurgery was used initially or subsequently at recurrence [15,16,25,32,33,34].

Of all patients, 29 patients died. Eight patients died from causes other than the underlying tumor disease, such as ischemic heart disease [35], urinary tract infection [36], pulmonary embolism [21], pneumonia [37,38], renal cell carcinoma [39], cerebellar hemorrhage related to anticoagulation [40], and other unrelated reasons [41].

### 3.4. Intermediate-Grade and Malignant Transformation

MM with a MIB–1/Ki-67 of 5–10% are defined as intermediate–grade MM with potential for development into malignant melanoma (>10% Ki-67). In total, 16 intermediate cases were reported, 14 showed a high recurrence rate without adjuvant radiotherapy after resection, and 2 were treated by radiotherapy without recurrence [42].

Furthermore, another 17 cases of MM reported malignant transformation [26,27,29,34,43,44]. Thirteen of these patients died, and four patients developed tumor progression. In 11 of these patients, MM metastases were found. All but three of these patients died of disease progression. Regarding the localization of metastasis, we were able to highlight that MM metastasize both within the central nervous system but also can occur in other tissues, as metastases were observed in the liver [8,22], pancreas [22], and skeleton [8] (Table 4).

Regarding the temporal component at follow–up, we were able to highlight a median of 18 months (range: few days–35 years).

## 4. Discussion

Since its first description in 1972 by Limas and Tio [2], several case descriptions of MM have been published; however, a meta-analysis of MM and evaluation of treatment strategies is missing but urgently needed. Our aim of this study was to analyze all available data from all MM cases published so far and evaluate potential treatment strategies.

Interestingly, our data demonstrated that MM occurs at a younger age in females than in males and is more frequent in males than in females, which contradicts the impression from smaller studies where female patients were thought to develop MM more often [50,51]. We could confirm the impression that MM is a disease of the adult, and in the majority of the cases independent of gender, MM occurred between the 3rd and 5th decade [51], while children were only rarely affected.

Furthermore, our analysis shows that the predominant location is the posterior fossa, as well as the cervical and thoracic spine, which is in line with the embryological development of melanocytes [52]. Melanocytes originate from the neural crest, which in turn gives rise, among other tissues, also to the leptomeninges. This may explain the preferred location of MM in the posterior fossa as well as the cervical and thoracic spine [52].

**Figure 4:**
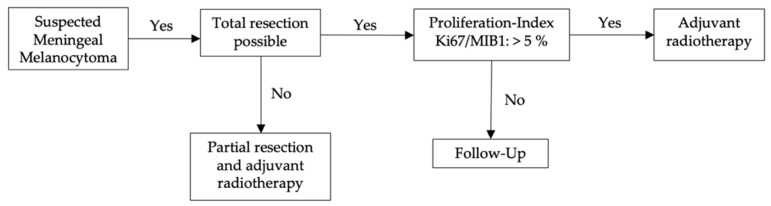
Treatment strategies at initial therapy: When MM is diagnosed, total resection should be the primary goal. Depending on the Ki–67 index, it should then be evaluated whether adjuvant radiotherapy is necessary.

We evaluated treatment strategies and their outcome, showing the best recurrence-free follow–up after total resection (Figure 4). In these cases, no clear evidence was found that adjuvant therapy is beneficial. Combined treatment with total resection and adjuvant radiotherapy may be considered only for those cases where tumors show high mitotic activity, i.e., intermediate MM, which has been observed to be associated with tumor recurrence. The differentiation to intermediate–grade (Ki67/MIB1–proliferation index of 5–10%) MM and malignant transformation into melanoma (Ki67/MIB1–proliferation index of >10%) is particularly important [53,54,55,56].

**Figure 5:**
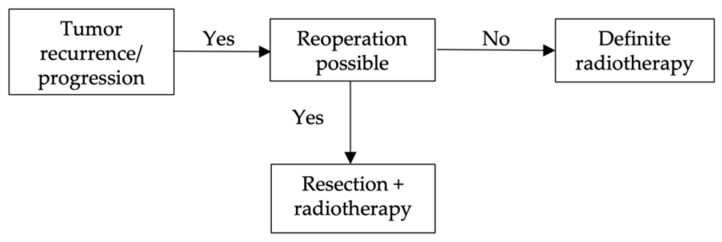
Treatment strategies after tumor recurrence/progression: Reoperation is the most used treatment option for tumor progression. However, in almost no case report, a second follow–up after the first recurrence is recorded, so no further evaluation can be made here as to how successful the respective therapy options are. These are only the therapies that are most frequently used.

Only in those cases that first received a total resection but developed MM recurrence, radiotherapy appears to be an advantage for recurrence-free survival (Figure 5).

In patients where only partial resection was achieved, radiotherapy showed a clear beneficial effect, while other therapeutic approaches, such as chemotherapy or immunotherapy, did not show any advantages. Furthermore, some case reports indicate a potential benefit of radiosurgery.

When MM is considered as a diagnosis, we do not suggest deciding on conservative follow-up observations, as we learned that MM appear capable of developing metastases. When the tumor is inoperable, definite radiotherapy has also been used successfully.

Therefore, we suggest the following treatment approach for patients that are diagnosed or considered to have MM (Figure 4):

Although chemo– and immunotherapy has failed so far, several mutations, such as in Guanine-nucleotide binding protein G subunit alpha (GNAQ, GNA11), have been identified [57]. Both GNAQ and GNA11 are g–proteins that share responsibility for activating the MAP–kinase pathway. Both have been suspected to be potential therapeutic targets. In mouse models, GNAQ and GNA11 have been shown to lead to hyperpigmentation and proliferation of melanocytes [58]. Additionally, molecular analysis of *PLCB4* and *CYSLTR2* [59,60,61] and methylation profiling are especially useful in discriminating these lesions from other pigmented CNS tumors [1,59]. The presence of *SF3B1*–, *EIF1AX*–, or *BAP1*–mutation or complex copy number variations indicate aggressive behavior consistent with meningeal melanoma [62,63,64]. Especially in children, meningeal melanocytosis and melanomatosis are characterized by NRAS- and occasionally BRAF mutations [65,66,67].

Beyond that, novel immunotherapies applied for malignant melanoma therapy may be beneficial also for MM. Both share common antigens, such as Melan-A or S–100 [11,12,13]. Therefore, further studies investigating novel potential therapies are needed.

### Limitations of the Study

As MM is a rare entity, the composition of our multi–center derived data set was very inhomogeneous due to different authors and unrelated case reports. For that reason, this study can only serve as a meta–analysis of all published case reports, which intuitively cannot fulfill the criteria for a real comprehensive study. Thus, future multi–center prospective studies with fixed follow-up periods are now needed in order to achieve precise outcome and prognosis and verify our treatment suggestions derived from our retrospective analysis.

The fact that we were unable to reanalyze the tissue samples in this study is a methodological limitation of studies such as this and can bias conclusions. Hopefully, future reports will be able to use more up–to–date histopathological classifications.

## 5. Conclusions

Based on the first retrospective meta–analysis of all MM cases published in the English language, we propose a novel guideline for the treatment of MM, highlighting the importance of total resection for recurrence-free follow-up and suggesting in which cases adjuvant radiotherapy may have beneficial effects of the patient’s disease course.

## Figures and Tables

**Table 2:** Total Resection and Follow–Up Data: Total resection alone showed the best outcome when it came to therapy options. Regarding adjuvant therapy, the data are very limited.

Therapy	Frequency	Follow–Up No Recurrence
Total Resection only	69 (90.8 %)	47/69 (68.1%)
Total Resection + RT	7 (9.2%)	5/7 (71.4%)
Total	76 (100%)	52/76 (68.4%)

**Table 3:** Partial Resection and Follow–Up Data: Partial Resection alone shows a high recurrence rate. The combination with adjuvant radiotherapy leads to more promising results.

Therapy	Frequency	Follow–Up No Recurrence
Partial Resection only	33 (55.9%)	18/33 (54.5%)
Partial Resection + RT	21 (35.6%)	13/21 (61.9%)
Partial Resection + Chemo	1 (1.7%)	1/1 (100%)
Partial Resection + RT + Chemo	2 (3.4%)	0/2 (0%)
Partial Resection + Radiosurgery	2 (3.4%)	2/2 (100%)
Total	59 (100%)	34/59 (57.6%)

**Table 4:** Metastasis: Metastasis led to either tumor progression or death. TR = Total Resection, PR = Partial Resection, RT = Radiotherapy, Reop = Reoperation.

Case	Primary Location MM	Location Metastasis	Therapy	Outcome
[8]	Spine	Liver, rib	TR, Reop + RT	Tumor progression
[17]	Intracranial	Thoracic	PR + RT, Reop + RT + Temolozomide + Cisplatin + Fotemustine	Death
[19]	Intracranial	Spine	TR, RT + Temozolomide	Death
[22]	Intracranial	Intracranial, Liver, pancreas	TR + RT, Reop + RT; Temozolomide; Ipilimumab;	Death
[27]	Spine	Intracranial	TR, Reop + RT + Pembrolizumab + Bevacizumab + Temozolomide	Death
[44]	Intracranial	Intracranial	TR, RT	Tumor progression
[45]	Spine	Intracranial	PR	Death
[46]	Spine	Intracranial	TR	Death
[47]	Spine	Spine	Resection	Death
[48]	Spine	Intracranial	PR, Reop + RT	Death
[49]	Spine	No data	TR, Reop	Tumor progression

## Data Availability

The data presented in this study are available in the supplementary material.

## Supplementary Materials

The following supporting information can be downloaded at: https://www.mdpi.com/article/10.3390/cancers14235851/s1, Table S1: All data of 201 case reports since 1972. RT = Radiotherapy; Surgery = Surgery without report of extent of resection. References [68,69,70,71,72,73,74,75,76,77,78,79,80,81,82,83,84,85,86,87,88,89,90,91,92,93,94,95,96,97,98,99,100,101,102,103,104,105,106,107,108,109,110,111,112,113,114,115,116,117,118,119,120,121,122,123,124,125,126,127,128,129,130,131,132,133,134,135,136,137,138,139,140,141,142,143,144,145,146,147,148,149,150,151,152,153,154,155,156,157,158,159,160,161,162,163,164,165,166,167,168,169,170,171,172,173,174,175,176,177,178,179,180,181,182,183,184,185,186,187,188,189,190,191,192,193,194,195,196,197,198] are mentioned in the supplementary materials.
