# Supplementary material for: How Should We Treat Meningeal Melanocytoma? A Retrospective Analysis of Potential Treatment Strategies
Source: Cancers (Basel). 2022 Nov 27;14(23):5851. doi: 10.3390/cancers14235851 (PMC9738837; doi:10.3390/cancers14235851)
Supplement: Supplementary file 1 [file cancers-14-05851-s001.zip › cancers-1978857-supplementary.pdf]

Supplementary Material

Table S1. This table lists 201 case reports that report patients with meningeal melanocytoma. Here, the focus was primarily on gender, age, location of the tumor, therapy, and outcome.

Case	Age	Sex	Location	Initial Therapy	Follow-Up Outcome
(A. Das et al., 2001)	50	M	Spine	Partial Resection + RT	Recurrence, Death
(Adib et al., 2019)	45	M	Intracranial	Partial Resection	No Data
(Adib et al., 2019)	54	M	Intracranial	Partial Resection	No Data
(Adib et al., 2019)	56	F	Intracranial	Surgery	No Data
(Adib et al., 2019)	33	F	Intracranial	Surgery	Recurrence
(Ahluwalia et al., 2003)	16	M	Intracranial	Total Resection	No Recurrence
(Aimar et al., 2003)	27	M	Intracranial	Surgery	No Data
(Aissaoui et al., 2015)	21	M	Intracranial	No Treatment	Death
(Akgun et al., 2020)	30	F	Spine	Total Resection	No Recurrence
(Al-Abdulwahhab et al., 2020)	23	M	Intracranial	Partial Resection	No Recurrence
(Alameda et al., 1998)	65	M	Spine	Total Resection	No Recurrence
(Albano et al., 2019)	51	M	Intracranial	Surgery	Recurrence
(Albano et al., 2019)	35	F	Intracranial	Total Resection	No Recurrence
(Ali et al., 2009)	31	M	Intracranial+Spine	Partial Resection	Recurrence, Death
(Armocida et al., 2019)	60	M	Spine	Total Resection	No Recurrence
(Asanuma et al., 2008)	29	M	Spine	Total Resection	No Recurrence
(Balmaceda et al., 1993)	-	-	No Data	No Data	No Data
(Balmaceda et al., 1993)	-	-	No Data	No Data	No Data
(Barth et al., 1993)	49	F	Spine	Surgery	Recurrence, Death
(Bejarano et al., 1995)	46	M	Spine	Total Resection	No Data
(Beseoglu et al., 2006)	55	M	Intracranial	Partial Resection + RT	No Recurrence
(Bhargava et al., 2011)	55	M	Spine	Partial Resection	No Data
(Bir et al., n.d.)	22	M	Intracranial	Total Resection + RT	No Recurrence
(Botticelli et al., 1983)	43	F	Intracranial	No Data	No Data
(Bourhis et al., 2019)	61	-	No Data	No Data	Recurrence
(Brito et al., n.d.)	-	-	No Data	Partial Resection	No Data
(Bydon et al., 2003)	79	F	Spine	Partial Resection	Recurrence, Death
(C. J. Chen et al., 1997)	41	F	Intracranial	Total Resection	No Recurrence
(C. Wang et al., 2022)	56	M	Spine	Partial Resection	No Recurrence
(Caruso et al., n.d.)	62	M	Spine	Total Resection	No Recurrence
(Châabane et al., 2003)	-	-	Intracranial	No Data	No Data
(Chacko & Rajshekhar, 2008)	22	M	Spine	Partial Resection	No Recurrence
(Chan et al., 2021)	18	F	Intracranial	Total Resection	No Recurrence
(Chow et al., 2001)	46	F	Intracranial	Total Resection	No Recurrence
(Clarke et al., 1998)	30	F	Intracranial	Partial Resection + RT	Recurrence, Death
(Clarke et al., 1998)	21	F	Spine	Partial Resection + RT	Recurrence
(Classen et al., 2002)	57	M	Intracranial	Definite Radiotherapy	No Recurrence
(Crouzet et al., 1992)	64	M	Spine	No Data	No Data
(Cusumano et al., 1998)	70	-	Intracranial	Surgery	Recurrence
(Czarnecki et al., n.d.)	69	F	Spine	Total Resection	No Data
(Czirják et al., 2000)	48	M	Intracranial	Total Resection	No Recurrence
(D. F. O'Brien et al., 2006)	10	-	Intracranial	Partial Resection	No Data
(de Tella et al., 2003)	35	M	Orbita	Partial Resection + RT + CT	Recurrence
(Delhaye et al., 2001)	38	F	Spine	Surgery	Recurrence, Death
(Deng et al., 2022)	19	F	Spine	Total Resection	Recurrence, Death
(Doglietto et al., 2012)	20	M	Intracranial	Partial Resection + RT	Recurrence, Death
(Donofrio et al., 2022)	48	F	Intracranial	Partial Resection + RT	No Recurrence
(Dorwal et al., 2014; Yin et al., 2019)	32	F	Spine	Total Resection	No Recurrence
(El-Khashab et al., 2009)	17	F	Spine	Partial Resection	No Data
(Elbadry et al., 2018)	46	M	Intracranial	Total Resection	No Data
(Eskandari & Schmidt, 2010)	45	M	Spine	Partial Resection	Recurrence
(Eun et al., 2011)	57	M	Spine	Total Resection	No Recurrence
(F. Wang & Ling, 2018)	32	F	Intracranial	Total Resection	No Recurrence
(F. Wang et al., 2007)	57	M	Spine	Total Resection	Recurrence
(F. Wang et al., 2011)	35	M	Intracranial	Total Resection	Recurrence
(Fagundes-Pereyra et al., 2005)	42	F	Intracranial	Total Resection	No Recurrence
(Fan et al., 2012)	48	M	Intracranial	No Data	No Data
(Faro et al., n.d.)	30	F	Intracranial	Total Resection	No Recurrence

(Fernandez et al., 2020)	67	M	Intracranial	Definite Radiotherapy	Recurrence, Death
(Ferracini et al., 1980)	46	F	Spine	Total Resection	Recurrence, Death
(Flores et al., 2021)	69	M	Spine	Partial Resection	No Data
(Foit et al., 2013)	43	-	Spine	Partial Resection + RT	No Recurrence
(Franken et al., 2009)	26	M	Intracranial	Partial Resection + RT	No Recurrence
(Gamoh et al., 2018)	39	M	Intracranial	Total Resection + RT	No Recurrence
(Ganesan et al., 2017)	34	M	Spine	Total Resection	No Data
(Gardiman' et al., 1996)	19	M	Intracranial	Partial Resection	No Recurrence
(Gardiman' et al., 1996)	43	M	Intracranial	Partial Resection	Recurrence, Death
(Gempt et al., 2011)	71	F	Intracranial	Total Resection	Recurrence
(Gill Naul et al., 1991)	68	F	Intracranial	Total Resection	No Recurrence
(Gonçjalves et al., 2002)	0	F	Spine	Total Resection	No Data
(González-Tortosa et al., 2009)	56	M	Intracranial	Total Resection	No Data
(Goyal et al., 2003)	33	F	Spine	Partial Resection + RT	No Data
(Gupta et al., 2007)	58	F	Intracranial	Partial Resection	No Recurrence
(H. Chen et al., 2015)	20	F	Intracranial	Total Resection	No Data
(Hamasaki et al., 2002)	59	M	Intracranial	Partial Resection	Recurrence
(Hean et al., 2021)	70	M	Spine	Partial Resection + RT + CT	Recurrence, Death
(Hino et al., 2005)	75	F	Intracranial	Partial Resection	No Recurrence
(Hioki et al., 2012)	72	M	Spine	Partial Resection	No Recurrence
(Hirose et al., 1997)	66	M	Intracranial	Total Resection	Recurrence, Death
(Hoffmann et al., 2016)	58	F	Spine	Total Resection + RT	No Data
(Horn et al., 2008)	48	M	Spine	Total Resection	Recurrence
(Horn et al., 2008)	37	F	Spine	Total Resection	Recurrence
(Horn et al., 2008)	37	F	Spine	Total Resection	Recurrence
(Hou et al., 2012)	-	-	No Data	Partial Resection + RT	No Recurrence
(Hou et al., 2012)	-	-	No Data	Partial Resection + RT	No Recurrence
(Hou et al., 2012)	-	-	No Data	Partial Resection + RT	No Recurrence
(Hou et al., 2012)	-	-	No Data	Partial Resection + RT	No Recurrence
(Hou et al., 2012)	-	-	No Data	Partial Resection + RT	Death
(Ibáñez et al., 1997)	44	F	Spine	Total Resection	No Recurrence
(Iida et al., 2002)	52	M	Spine	Partial Resection	No Recurrence
(Iida et al., 2002)	42	M	Spine	Surgery	Recurrence, Death
(J. K. Lee et al., 2017)	45	M	Spine	Total Resection	No Recurrence
(Jellinger et al., 1988)	27	F	Intracranial	Total Resection	Recurrence
(Ju-Sung Seo, n.d.)	58	M	Spine	Total Resection	No Recurrence
(K. Das et al., 2017)	17	M	Intracranial	Total Resection	No Recurrence
(K. T. K. Chen, n.d.)	65	F	Spine	Partial Resection + RT	No Data
(Kang et al., 2006)	29	M	Intracranial	Partial Resection	No Data
(Khadilkar et al., n.d.)	58	F	Spine	No Data	No Data
(Kim et al., 2013)	37	M	Spine	Partial Resection + RT	Recurrence, Death
(Kini et al., 2009)	35	M	Intracranial	Total Resection	Recurrence
(Kinnen et al., 2019)	64	M	Spine	Partial Resection	No Recurrence
(Koch et al., 2005)	38	M	Intracranial	Total Resection	Recurrence, Death
(Koenigsmann et al., n.d.)	57	M	Intracranial	Total Resection	Recurrence, Death
(Kordás et al., 1996)	12	F	Spine	Total Resection + RT	No Recurrence
(Kraft Roverea et al., 2014)	54	F	Spine	No Data	No Data
(Kuo et al., 2019)	51	F	Intracranial	Total Resection	Recurrence
(Kurita et al., 2000)	29	M	Intracranial	Partial Resection	Recurrence
(Küsters-Vandevelde et al., 2017)	43	F	Intracranial	Total Resection + RT	Recurrence, Death
(Lach et al., 1988)	20	F	Spine	Total Resection	No Recurrence
(Leonardi1 et al., n.d.)	67	M	Intracranial	Total Resection	No Recurrence
(Limas & Tio, 1972)	71	M	Intracranial	No Treatment	Death
(Lin et al., 2012)	27	M	Intracranial	Total Resection + RT	No Recurrence
(Litofsky et al., 1992)	32	M	Intracranial	Total Resection	No Recurrence
(Maaloul et al., 2021)	30	M	Intracranial	No Treatment	Recurrence
(Maiuri et al., 1995)	69	M	Intracranial	Partial Resection	No Recurrence
(Malik et al., 1999)	48	M	Spine	Total Resection	No Recurrence
(Mangels et al., 2006)	35	F	Spine	Partial Resection + RT	No Recurrence
(Marwaha et al., 2016)	21	M	Intracranial	Partial Resection + RT	No Recurrence
(Mathai et al., 2008)	40	M	Orbita	Total Resection	No Recurrence
(Matsumoto et al., 1998)	48	M	Spine	Total Resection	No Recurrence
(Matsuno et al., 2019)	35	F	Intracranial	Partial Resection	No Recurrence
(Merciadri et al., 2011)	68	M	Intracranial+Spine	Partial Resection + RT	No Recurrence
(Mitchell & Funt, 1998)	49	M	Intracranial	No Treatment	No Data

(Miura et al., 2020)	40	M	Spine	Total Resection	No Recurrence
(Munoz-Hidalgo, 2014)	15	M	Intracranial	Total Resection	No Recurrence
(Muthappan et al., 2012)	61	F	Spine	Total Resection	No Recurrence
(N. K. Lee et al., 2015)	15	F	Intracranial	Partial Resection + Radiosurgery	No Recurrence
(Nakahara et al., 2010)	0,5	M	Intracranial	No Treatment	Death
(Navas et al., 2009)	25	M	Intracranial	Total Resection	Death
(Nicolotto et al., 2018)	-	-	Spine	Surgery + CT	No Data
(Offiah & Laitt, n.d.)	25	F	Intracranial	Total Resection	No Recurrence
(Oruckaptan et al., 2000)	0,5	M	Intracranial	Partial Resection	No Recurrence
(Painter et al., 2000)	35	M	Intracranial	Partial Resection	No Data
(Palicelli et al., 2016)	26	M	Orbita	Total Resection	No Recurrence
(Pan et al., 2011)	36	M	Intracranial	Partial Resection + RT	No Recurrence
(Perrini et al., 2010)	79	F	Spine	Partial Resection	Recurrence
(Phang et al., 2012)	40	M	Intracranial	Partial Resection + RT	Recurrence, Death
(Piercecchi-Marti et al., 2002)	25	M	Intracranial	Total Resection	No Recurrence
(Pierson et al., 2016)	22	M	Intracranial	Partial Resection + Radiosurgery	No Recurrence
(Prabhu et al., 1993)	67	F	Intracranial	Surgery	No Recurrence
(Prabhu et al., 1993)	27	F	Intracranial	Total Resection + RT	No Recurrence
(Rades et al., 2001)	23	F	Spine	Partial Resection	Recurrence, Death
(Rahimi-Movaghar & Karimi, 2003)	17	M	Intracranial	Total Resection	No Recurrence
(Rai et al., 2008)	35	M	Intracranial	Total Resection	No Data
(Reddy et al., 2012)	43	M	Spine	Total Resection	No Recurrence
(Ren et al., 2015)	29	F	Intracranial	Partial Resection + RT	No Recurrence
(Roser et al., 2004)	37	F	Intracranial	Partial Resection	Recurrence, Death
(Rousseau et al., 2005)	25	F	Intracranial	Partial Resection	No Recurrence
(Ruelle et al., 1996)	62	M	Spine	Total Resection	No Recurrence
(Rutten et al., 2005)	37	F	Orbita	Total Resection	Recurrence
(S.-U. Lee et al., 2018)	22	F	Intracranial	Surgery	No Data
(Sakar et al., 2022)	1,5	F	Intracranial	Total Resection	No Recurrence
(Sakata et al., 2015)	31	F	Intracranial	Partial Resection	Recurrence
(Salah El-Din et al., 2018)	5	M	Spine	Partial Resection	No Data
(Samadian et al., 2015)	19	M	Intracranial	Total Resection	No Recurrence
(Samimi et al., 2012)	38	M	Intracranial	Partial Resection	Recurrence
(San-Miguel et al., 2020)	46	F	Intracranial	Total Resection	Recurrence, Death
(Sankhla et al., 1996)	19	M	Spine	Partial Resection	No Recurrence
(Sato et al., 2009)	49	M	Orbita	Partial Resection + CT	No Recurrence
(Schembri et al., 2017)	53	M	Spine	Surgery	No Data
(Schindler et al., 1998)	38	M	Intracranial	Total Resection	No Recurrence
(Sethi et al., 2011)	40	M	Spine	Surgery	No Data
(Shaikh et al., 2021)	36	F	Spine	Total Resection	Recurrence, Death
(Shanthi et al., 2010)	30	F	Spine	Total Resection	No Data
(Sharma et al., n.d.)	26	F	Spine	Partial Resection + RT	No Data
(Shimoda et al., n.d.)	75	F	Spine	Partial Resection	No Data
(Shin et al., 2020)	56	F	Intracranial	Surgery + RT	No Data
(Shinoda et al., 2000)	40	F	Intracranial	Total Resection	No Recurrence
(Shownkeen et al., 2002)	38	M	Spine	Surgery	No Data
(Srirama Jayamma et al., 2015)	62	M	Spine	Surgery	No Data
(Steinberg et al., 1978)	71	F	Spine	Partial Resection + RT	Recurrence
(Sun et al., 2015)	56	M	Intracranial	Partial Resection	Recurrence
(Sung Hye Park et al., 1992)	41	F	Spine	Partial Resection	No Data
(T. F. O'Brien et al., 1995)	71	F	Intracranial	No Data	No Data
(T. F. O'Brien et al., 1995)	39	-	Intracranial	No Data	No Data
(T. F. O'Brien et al., 1995)	40	-	Intracranial	No Data	No Data
(T. F. O'Brien et al., 1995)	49	-	Intracranial	No Data	No Data
(Tatagiba et al., 1992)	40	M	Spine	Total Resection	No Data
(Tateyama et al., 2020)	27	M	Spine	Partial Resection	Recurrence
(Tewari et al., 1990)	-	-	No Data	No Data	No Data
(Tewari et al., 1990)	-	-	No Data	No Data	No Data
(Tregnago et al., 2015)	28	M	Orbita	Total Resection	No Recurrence
(Tsai et al., 2021)	33	M	Spine	Total Resection	Recurrence
(Tsugu et al., 2009)	51	M	Orbita	Total Resection	No Recurrence
(Turhan et al., 2004)	-	-	No Data	Total Resection	No Data
(Turhan et al., 2004)	-	M	Spine	Total Resection	No Data

(Uematsu et al., 1992)	62	M	Intracranial	Total Resection	No Recurrence
(Uozumi et al., 2003)	49	M	Intracranial	Total Resection	Recurrence, Death
(URAMARU et al., 2021)	44	M	Intracranial	Total Resection	No Recurrence
(Vaidya et al., 2021)	29	F	Spine	Partial Resection	Recurrence
(Verma et al., 1979)	71	F	Spine	Total Resection + RT	Recurrence
(Vreto et al., 2011)	47	M	Intracranial	Surgery	No Data
(Winston et al., 1987)	9	M	Intracranial	Total Resection	Recurrence
(Xie & Jiang, 2019)	73	M	Spine	Total Resection	No Recurrence
(Yang et al., 2016)	41	F	Spine	Partial Resection	Recurrence, Death
(Yin et al., 2019)	32	F	Spine	Total Resection	No Recurrence
(Yukawa et al., 2003)	29	F	Intracranial	Total Resection	No Recurrence
(Zeiler & Krcek, 2012)	25	F	Intracranial	Partial Resection	No Recurrence
(Zhi-Qiang Liu, 2021)	52	M	Spine	Partial Resection	No Recurrence
(Zhou et al., 2021)	32	M	Orbita	Radiosurgery	Recurrence
Our Case	25	M	Intracranial	Total Resection	Recurrence

References

1. Das, A.; Ratnagopal, P.; Puvanendran, K.; Teo, J.G. Spinal Meningeal Melanocytoma with Hydrocephalus and Intracranial Superficial Siderosis. *Intern. Med. J.* **2001**, *31*, 562–564. https://doi.org/10.1046/j.1445-5994.2001.00119.x.
2. Adib, S.D.; Ebner, F.H.; Bornemann, A.; Hempel, J.-M.; Tatagiba, M. Surgical Management of Primary Cerebellopontine Angle Melanocytoma: Outcome, Recurrence and Additional Therapeutic Options. World Neurosurg. **2019**, *128*, e835–e840. https://doi.org/10.1016/j.wneu.2019.05.004.
3. Ahluwalia, S.; Ashkan, K.; Casey, A.T.H. Meningeal Melanocytoma: Clinical Features and Review of the Literature. Br. J. Neurosurg. **2003**, *17*, 347–351. https://doi.org/10.1080/02688690310001601243.
4. Aimar, E.; Debernardi, A.; Tancioni, F.; di Ieva, A.; Bossi, P.; Gaetani, P.; Rodriguez y Baena, R. Meningeal Melanocytoma of the Temporal Lobe. An Uncommon Tumor in an Unusual Location. Case Report. J. Neurosurg. Sci. **2003**, *47*, 211.
5. Aissaoui, A.; Mosrati, M.A.; Moussa, A.; Belhaj, M.; Bougattas, M.; Zakhama, A.; Chadly, A. Sudden Death and Primary Leptomeningeal Melanocytosis: A Case Report With an Autopsy Diagnosis. Am. J. Forensic. Med. Pathol. **2015**, *36*, 199–201. https://doi.org/10.1097/PAF.0000000000000161.
6. Akgun, M.Y.; Isler, C.; Ulu, M.O. C6-T1 Intradural Extramedullary Ventral Meningeal Melanocytoma Resected Via Anterior Corpectomy with Reconstruction. World Neurosurg. **2020**, *138*, 457–460. https://doi.org/10.1016/j.wneu.2020.03.122.
7. Al-Abdulwahhab, A.H.; Al-Sharydah, A.M.; Al-Suhibani, S.S.; Al-Shayji, H.; Al-Saad, I.; Al-Issawi, W. Primary Pigmented Meningeal Melanocytoma Originating in Meckel Cave in a Patient with Carney Complex: A Case Report. Medicine **2020**, *99*, e18783. https://doi.org/10.1097/MD.0000000000018783.
8. Alameda, F.; Lloreta, J.; Galitó, E.; Roquer, J.; Serrano, S. Meningeal Melanocytoma: A Case Report and Literature Review. Ultrastruct. Pathol. **1998**, *22*, 349–356. https://doi.org/10.3109/01913129809103356.
9. Albano, L.; Losa, M.; Spatola, G.; Panni, P.; Terreni, M.R.; Barzaghi, L.R.; Mortini, P. Primary Sellar Melanocytoma: Report of Two Cases Treated at the Same Institution and Their Long-Term Outcome. Br. J. Neurosurg. 2019 . https://doi.org/10.1080/02688697.2019.1667485.
10. Albano, L.; Losa, M.; Spatola, G.; Panni, P.; Terreni, M.R.; Barzaghi, L.R.; Mortini, P. Primary Sellar Melanocytoma: Report of Two Cases Treated at the Same Institution and Their Long-Term Outcome. Br. J. Neurosurg. 2019 . https://doi.org/10.1080/02688697.2019.1667485.
11. Ali, Y.; Rahme, R.; Moussa, R.; Abadjian, G.; Menassa-Moussa, L.; Samaha, E. Multifocal Meningeal Melanocytoma: A New Pathological Entity or the Result of Leptomeningeal Seeding? J. Neurosurg. **2009**, *111*, 488–491. https://doi.org/10.3171/2009.3.JNS081096.
12. Armocida, D.; Pesce, A.; Berra, L.V.; Marzetti, F.; Antonelli, M.; Santoro, A. Intradural Extramidullary Dorsal Melanocytoma in the Adult: Case Report and Review of the Literature. J. Clin. Neurosci. **2019**, *62*, 248–253. https://doi.org/10.1016/j.jocn.2018.12.004.
13. Asanuma, K.; Kasai, Y.; Takegami, K.; Ito, H.; Yoshikawa, T.; Uchida, A. Spinal Neurocutaneous Melanosis without Cutaneous Nevi. Spine (Phila Pa 1976) **2008**, *33*, E798–E801. https://doi.org/10.1097/BRS.0b013e3181808c9a.
14. Balmaceda, C.M.; Fetell, M.R.; O'Brien, J.L.; Housepian, E.H. Nevus of Ota and Leptomeningeal Melanocytic Lesions. Neurology **1993**, *43*, 381. https://doi.org/10.1212/wnl.43.2.381.
15. Barth, A.; Pizzolato, G.P.; Berney, J. Intramedullary Meningeal Melanocytoma. *Neurochirurgie* **1993**, *39*, 188–194.
16. Bejarano, B.; Isla, A.; Morales, C.; Paz, J.; Blázquez, M.G. Melanocitoma Meníngeo Espinal. Neurocirugia **1995**, *6*, 230–232. https://doi.org/10.1016/S1130-1473(95)70785-0.
17. Beseoglu, K.; Knobbe, C.B.; Reifenberger, G.; Steiger, H.-J.; Stummer, W. Supratentorial Meningeal Melanocytoma Mimicking a Convexity Meningioma. Acta Neurochir. **2006**, *148*, 485–490. https://doi.org/10.1007/s00701-005-0705-1.
18. Bhargava, P.; Grewal, S.S.; Dewan, Y.; Jhawar, S.S.; Jain, V.; Gupta, B. Craniovertebral Junction Melanocytoma: A Case Report. Turk. Neurosurg. 2011 . https://doi.org/10.5137/1019-5149.jtn.5272-11.0.

19. Bir, H.; Sodhi, S.; Salunke, P.; Sahoo, S.K.; Radotra, B.D.; Kumar, N. Primary Ventral Foramen Magnum Meningeal Melanocytoma. Neurology **2014**, *62*, 230.
20. Botticelli, A.R.; Villani, M.; Angiari, P.; Peserico, L. Meningeal Melanocytoma of Meckel's Cave Associated with Ipsilateral Ota's Nevus. Cancer **1983**, *51*, 2304–2310. https://doi.org/10.1002/1097-0142(19830615)51:12<2304::aid-cncr2820511223>3.0.co;2-u.
21. Bourhis, A.; Quintin-Roué, I.; Redon, S.; Bourhis, M.; Magro, E.; Seizeur, R.; Marcorelles, P.; Uguen, A. Transformation d'un Mélanocytome Méningé En Mélanome : Étude Clinique, Histopathologique et Cytogénétique. Ann. Pathol. **2019**, *39*, 352–356. https://doi.org/10.1016/j.annpat.2019.01.010.
22. Brito, A.B.; Rogerio, F.; Reis, F.; Garmes, H.M.; Vassallo, J.; Lima, C.S. Primary Meningeal Melanocytoma Mimicking a Non-functioning Pituitary Adenoma. Clin. Neuropathol. **2016**, *35*, 158. https://doi.org/10.5414/NP300885.
23. Bydon, A.; Gutierrez, J.A.; Mahmood, A. Meningeal Melanocytoma: An Aggressive Course for a Benign Tumor. *J. Neurooncol.* **2003**, *64*, 259–263. https://doi.org/10.1023/a:1025628802228.
24. Chen, C.J.; Hsu, Y.I.; Ho, Y.S.; Hsu, Y.H.; Wang, L.J.; Wong, Y.C. Intracranial Meningeal Melanocytoma: CT and MRI. Neuroradiology **1997**, *39*, 811–814. https://doi.org/10.1007/s002340050510.
25. Wang, C.; Shao, X.; Zou, Y. Primary Intramedullary Melanocytoma in the Thoracic Cord: A Case Report and Literature Review. Transl. Cancer Res. **2022**, *11*, 928–934. https://doi.org/10.21037/tcr-21-2132.
26. Caruso, R.; Marrocco, L.; Wierzbicki, V.; Salvati, M. Intramedullary Melanocytoma: Case Report and Review of Literature. Tumori **2009**, *95*, 389–393.
27. Châabane, M.; Ellouze, S.; Hamrouni, A.; Mlika, N.; ben Hammouda, M.; Khoudja, F. Meningeal Melanocytoma: A Rare Meningeal Tumor. J. Radiol. **2003**, *84*, 415–416.
28. Chacko, G.; Rajshekhar, V. Thoracic Intramedullary Melanocytoma with Long-Term Follow-up: Case Report. J. Neurosurg. Spine **2008**, *9*, 589–592. https://doi.org/10.3171/SPI.2008.9.08323.
29. Chan, I.Y.M.; Li, H.; Shi, T.; Hammond, R.R.; Jurkiewicz, M.T. Parietal Convexity Meningeal Melanocytoma: Radiolog-ic-Pathologic Correlation. Can. J. Neurol. Sci. **2021**, *48*, 719–721. https://doi.org/10.1017/cjn.2020.256.
30. Chen, H.; Liu, W.; Zhang, S.; Xu, J.; Hui, X. Cerebellar Meningeal Melanocytoma Associated with Nevus of Ota: An Extremely Rare Case. Neurology **2015**, *85*, 555–556.
31. Chen, K.T.K. Crush Cytology of Melanocytoma of the Spinal Cord. A Case Report. Acta Cytol. **2003**, *47*, 1091–1094. https://doi.org/10.1159/000326654.
32. Chow, M.; Clarke, D.B.; Maloney, W.J.; Sangalang, V. Meningeal Melanocytoma of the Planum Sphenoidale. Case Report and Review of the Literature. J. Neurosurg. **2001**, *94*, 841–845. https://doi.org/10.3171/jns.2001.94.5.0841.
33. Clarke, D.B.; Leblanc, R.; Bertrand, G.; Quartey, G.R.; Snipes, G.J. Meningeal Melanocytoma. Report of a Case and a Historical Comparison. *J. Neurosurg.* **1998**, *88*, 116–121. https://doi.org/10.3171/jns.1998.88.1.0116.
34. Classen, J.; Hehr, T.; Paulus, W.; Plate, K.; Bamberg, M. Suprasellar Melanocytoma: A Case of Primary Radiotherapy and Review of the Literature. *J. Neuro-Oncol.* **2002**, *58*, 39–46.
35. Crouzet, J.; Richard, S.; Pillet, G.; Muckensturm, B.; Srour, A.; Pradat, P. Meningeal Melanocytoma or Multiple Pigmented Meningioma of the Spinal Canal. Report of a Case. Review of the Literature. Rev. Rhum. Mal. Osteoartic. **1992**, *59*, 738–743.
36. Cusumano, S.; Marchiofi, G.; Trincia, G.; Barotto, V.; Tonetto, G. Malignant Meningeal Melanoma; Springer-Verlag : 1998; Volume 19.
37. Czarnecki, E.J.; Silbergleit, R.; Gutierrez, J.A. MR of Spinal Meningeal Melanocytoma. Am. J. Neuroradiol. **1997**, *18*, 180–182.
38. Czirják, S.; Vitanovic, D.; Slowik, F.; Magyar, A. Primary Meningeal Melanocytoma of the Pineal Region. Case Report. J. Neurosurg. **2000**, *92*, 461–465. https://doi.org/10.3171/jns.2000.92.3.0461.
39. O'Brien, D.F.; Crooks, D.; Mallucci, C.; Javadpour, M.; Williams, D.; du Plessis, D.; Broome, J.; Foy, P.; Pizer, B. Meningeal Melanocytoma. Childs Nerv. Syst. **2006**, *22*, 556–561. https://doi.org/10.1007/s00381-005-0019-x.
40. de Tella, O.I.; Agner, C.; Aguiar, P.H.; Herculano, M.A.; Prandini, M.N.; Stavile, J.N. Aggressive Management of Orbital Meningeal Melanocytoma. *Acta Neurochir.* **2003**, *145*, 1121–1126. https://doi.org/10.1007/s00701-003-0121-3.
41. Delhaye, M.; Menei, P.; Rousselet, M.C.; Diabira, S.; Mercier, P. A Case of Intramedullary Primary Melanocytic Tumor: Meningeal Melanocytoma or Malignant Melanoma? Neurochirurgie **2001**, *47*, 133–136.
42. Deng, S.L.; Wang, Y.B.; Wang, D.H.; Zhan, S.; Jing, Y.; Guan, Y. Malignant Transformation and Metastatic Spread of Dumbbell-Shaped Meningeal Melanocytoma of the Cervical Spine: A Case Report and Literature Review. *Front. Surg.* **2022**, *9*, 789256. https://doi.org/10.3389/fsurg.2022.789256.
43. Doglietto, F., Colosimo, C., Lauriola, L. et al. Intracranial melanocytic meningeal tumours and melanosis oculi: case report and literature review. *BMC Cancer* **2012**, 12, 220. https://doi.org/10.1186/1471-2407-12-220;.
44. Donofrio, C.A.; Roncaroli, F.; Riccio, L.; Pereira, M.; O'Sullivan, J.; Mayers, H.; Potter, G.M.; Djoukhadar, I.; Rutherford, S.A. A Challenging Case of Sporadic Melanocytoma of the Jugular Foramen. Neurochirurgie **2021**, *68*, 453–457. https://doi.org/10.1016/j.neuchi.2021.06.001.
45. Dorwal, P.; Gautam, D.; Mohapatra, I.; Gupta, A. Intramedullary Melanocytoma of Thoracic Spine: A Rare Case Report. Asian J. Neurosurg. **2014**, *9*, 36. https://doi.org/10.4103/1793-5482.131068.
46. El-Khashab, M.; Koral, K.; Bowers, D.C.; Johnson-Welch, S.; Swift, D.; Nejat, F. Intermediate Grade Meningeal Melanocytoma of Cervical Spine. *Childs Nerv. Syst.* **2009**, *25*, 407–410. https://doi.org/10.1007/s00381-008-0782-6.

47. Elbadry, R.; Elazim, A.A.; Mohamed, K.; Issa, M.; Ayyad, A. Primary Meningeal Melanocytoma of the Cerebellopontine Angle Associated with Ipsilateral Nevus of Ota: A Case Report. Surg. Neurol. Int. **2018**, *9*, 245. https://doi.org/10.4103/SNI.SNI_235_18.
48. Eskandari, R.; Schmidt, M.H. Intramedullary Spinal Melanocytoma. Rare Tumors **2010**, *2*, 64–67. https://doi.org/10.4081/rt.2010.e24.
49. Eun, S.S.; Kim, H.S.; Lee, S.H.; Liu, W.C.; Lee, J.H. Spinal Meningeal Melanocytoma in the S-1 Nerve Root Sheath with Paraspinal Extension Mimicking Schwannoma. World Neurosurg. **2011**, *75*, 303–306.
50. Wang, F.; Ling, S. Primary Meningeal Melanocytoma in Sellar Region, Simulating a Nonfunctioning Pituitary Adenoma: Case Report and Literature Review. World Neurosurg. **2018**, *112*, 209–213. https://doi.org/10.1016/j.wneu.2018.01.145.
51. Wang, F.; Li, X.; Chen, L.; Pu, X. Malignant Transformation of Spinal Meningeal Melanocytoma. Case Report and Review of the Literature. *J. Neurosurg. Spine* **2007**, *6*, 451–454. https://doi.org/10.3171/spi.2007.6.5.451.
52. Wang, F.; Qiao, G.; Lou, X.; Song, X.; Chen, W. Malignant Transformation of Intracranial Meningeal Melanocytoma. Case Report and Review of the Literature. Neuropathology **2011**, *31*, 414–420. https://doi.org/10.1111/j.1440-1789.2010.01160.x.
53. Fagundes-Pereyra, W.J.; de Sousa, L.; Carvalho, G.T.C.; Pittella, J.E.H.; de Sousa, A.A. Meningeal Melanocytoma of the Posterior Fossa: Case Report and Literature Review. *Surg. Neurol.* **2005**, *63*, 269–274. https://doi.org/10.1016/j.surneu.2004.05.035.
54. Fan, M.; Wang, J.; Fu, W.; Liu, K.; Li, L.; Sun, P. Primary Meningeal Melanocytoma Located in Foramen Magnum: A Case Report and Review of the Literatures. Chin. Med. Sci. J. **2012**, *27*, 115–120.
55. Faro, S.H.; Koenigsberg, R.A.; Turtz, A.R.; Croul, S.E. Melanocytoma of the Cavernous Sinus: CT and MR Findings. Am. J. Neuroradiol. **1996**, *17*, 1087–1090.
56. Fernandez, C.; Hoeltzel, G.; Werner-Wasik, M.; Kenyon, L.C.; Shi, W. Definitive Radiotherapy for Meningeal Brainstem Melanocytoma: A Case Report. *Br. J. Neurosurg.* **2020**, 1-4. https://doi.org/10.1080/02688697.2020.1864291.
57. Ferracini, R.; Gardini, G.; Brisigotti, M.; Lanzanova, G.; Manetto, V.; Lorenzini, P. Metastasizing Meningeal Melanocytoma. *Tumori* **1980**, *66*, 405–408.
58. Flores, A.; Gadot, R.; Noorbhai, I.; Hall, H.; Heck, K.A.; Raper, D.M.S.; Xu, D.; Karas, P.; Mandel, J.J.; Ropper, A.E. S-100-Negative, GNA11 Mutation-Positive Intramedullary Meningeal Melanocytoma of the Thoracic Spine: A Radiographic Challenge and Histologic Anomaly. Surg. Neurol. Int. **2021**, *12*, 315.
59. Foit, N.A.; Neidert, M.C.; Woernle, C.M.; Rushing, E.J.; Krayenbü Hl, N. Bifocal Extra- and Intradural Melanocytoma of the Spine: Case Report and Literature Review. Eur. Spine J. **2013**, *22*, 521–525.
60. Franken, S.P.G.; Setz-Pels, W.; Smink-Bol, M.; Gijtenbeek, J.M.M.; Nanda, D.; van der Maazen, R.W.M.; van der Vliet, T.; Bussink, J. Unusual Case of Bifocal Leptomeningeal Melanocytoma in the Posterior Fossa with Seeding in the Spinal Canal. Br. J. Radiol. **2009**, *82*, e182–e188. https://doi.org/10.1259/bjr/30756805.
61. Gamoh, S.; Tsuno, T.; Akiyama, H.; Kotaki, S.; Nakanishi, T.; Tsuji, K.; Yoshida, H.; Shimizutani, K. Intracranial Meningeal Melanocytoma Diagnosed Using an Interdisciplinary Approach: A Case Report and Review of the Literature. J. Med. Case Rep. **2018**, *12*, 177. https://doi.org/10.1186/s13256-018-1725-9.
62. Ganesan, S.; Acharya, S.; Kalra, K.; Chahal, R. Intradural Intramedullary Primary Spinal Melanocytoma: A Rare Case Report. Indian J. Neurosurg. **2017**, *6*, 055–058. https://doi.org/10.1055/s-0036-1584589.
63. Gardiman, M.; Altavilla, G.; Marchioro, L.; Boscolo, L.; Alessio, L.; Piazza, M. Meningeal Melanocytoma: A Rare Lesion of The Central Nervous System. *Tumori J.* **1996**, *82*, 494–496.
64. Gempt, J.; Buchmann, N.; Grams, A.E.; Zoubaa, S.; Schlegel, J.; Meyer, B.; Ringel, F. Black Brain: Transformation of a Melanocytoma with Diffuse Melanocytosis into a Primary Cerebral Melanoma. *J. Neurooncol.* **2011**, *102*, 323–328. https://doi.org/10.1007/s11060-010-0311-9.
65. Gill Naul, L.; Hise, J.H.; Bauserman, S.C.; Todd, F.D. CT and MR of Meningeal Melanocytoma; *AJNR Am J Neuroradiol* **1991**, 315-316.
66. Gonçjalves, J.; Díaz, P.; Maíllo, A.; Blanco, A.; Subhi-Issa, I. Melanocitoma Meníngeo Cervical Simulando Un Neurinoma de La Raíz C7: Caso Clínico. Neurocirugia **2002**, *13*, 393–396. https://doi.org/10.1016/S1130-1473(02)70595-2.
67. González-Tortosa, J.; Ros de San Pedro, J.; Ferri-Ñíguez, B. Melanocitoma Meníngeo Del Ángulo Pontocerebeloso: ¿Un Tumor Benigno? Neurocirugia **2009**, *20*, 372–379. https://doi.org/10.1016/S1130-1473(09)70157-5.
68. Goyal, A.; Sinha, S.; Singh, A.K.; Tatke, M.; Kansal, A. Lumbar Spinal Meningeal Melanocytoma of the L3 Nerve Root with Paraspinal Extension: A Case Report. Spine (Phila Pa 1976) **2003**, *28*, E140–E142. https://doi.org/10.1097/01.BRS.0000051879.20360.8A.
69. Gupta, A.; Ahmad, F.U.; Sharma, M.C.; Garg, A.; Mehta, V.S. Cerebellopontine Angle Meningeal Melanocytoma: A Rare Tumor in an Uncommon Location. Case Report. J. Neurosurg. **2007**, *106*, 1094–1097. https://doi.org/10.3171/jns.2007.106.6.1094.
70. Hamasaki, O.; Nakahara, T.; Sakamoto, S.; Kutsuna, M.; Sakoda, K. Intracranial Meningeal Melanocytoma-Case Report. *Neurol. Med. -Chir.* **2002**, *42*, 504–509.
71. Hean, V.; Bouleftour, W.; Ramirez, C.; Forest, F.; Boutet, C.; Rivoirard, R. Nivolumab as Adjuvant Treatment for a Spinal Melanocytoma: A Case Report. *Medicine* **2021**, *100*, e25862. https://doi.org/10.1097/MD.0000000000025862.
72. Hino, K.; Nagane, M.; Fujioka, Y.; Shiokawa, Y. Meningeal Melanocytoma Associated with Ipsilateral Nevus of Ota Presenting as Intracerebral Hemorrhage: Case Report. *Neurosurgery* **2005**, *56*, E1376. https://doi.org/10.1227/01.neu.0000159716.45457.bc.

73. Hioki, A.; Miyamoto, K.; Kato, H.; Hatano, Y.; Asano, N.; Hirose, Y.; Fushimi, K.; Shimizu, K. Sudden Onset of Paraplegia Caused by Subarachnoid Hemorrhage Associated with Meningeal Melanocytoma of the Conus Medullaris: A Case Report of Intraoperative Identification of These Two Pathological Conditions. Eur. J. Orthop. Surg. Traumatol. **2012**, *22*, 593–596.
74. Hirose, T.; Horiguchi, H.; Kaneko, F.; Kusaka, K.; Morizumi, H.; Seki, K.; Sano, T. Melanocytoma of the Foramen Magnum. *Pathol. Int.* **1997**, *47*, 155–160. https://doi.org/10.1111/j.1440-1827.1997.tb03733.x.
75. Hoffmann, M.; Koelsche, C.; Seiz-Rosenhagen, M.; Mai, S.; Lohr, F.; Reuss, D.; Wenz, F.; Gebhardt, C.; Giordano, F.A. The GNAQ in the Haystack: Intramedullary Meningeal Melanocytoma of Intermediate Grade at T9-10 in a 58-Year-Old Woman. J. Neurosurg. **2016**, *125*, 53–56. https://doi.org/10.3171/2015.5.JNS15233.
76. Horn, E.M.; Nakaji, P.; Coons, S.W.; Dickman, C.A. Surgical Treatment for Intramedullary Spinal Cord Melanocytomas. *J. Neurosurg. Spine* **2008**, *9*, 48–54. https://doi.org/10.3171/SPI/2008/9/7/048.
77. Hou, G.Q.; Sun, J.C.; Zhang, X.J.; Shen, B.X.; Zhu, X.J.; Liang, L.; Zhang, X.L. MR Imaging Findings of the Intraspinal Meningeal Melanocytoma: Correlation with Histopathologic Findings. Am. J. Neuroradiol. **2012**, *33*, 1525–1529. https://doi.org/10.3174/ajnr.A2987.
78. Ibáñez, J.; Weil, B.; Ayala, A.; Jimenez, A.; Acedo, C.; Rodrigo, I. Meningeal Melanocytoma: Case Report and Review of the Literature. *Histopathology* **1997**, *30*, 576–581. https://doi.org/10.1046/j.1365-2559.1997.5660798.x.
79. Iida, M.; Llena, J.F.; Suarez, M.A.; Malik, S.; Weidenheim, K.M.; LaSala, P.; Hirano, A. Two Cases of Spinal Meningeal Melanocytoma. Brain Tumor Pathol. **2002**, *19*, 41–45. https://doi.org/10.1007/BF02482455.
80. Lee, J.K.; Rho, Y.J.; Jeong, D.M.; Rhim, S.C.; Kim, S.J. Diagnostic Clue of Meningeal Melanocytoma: Case Report and Review of Literature. Yonsei Med. J. **2017**, *58*, 467–470.
81. Jellinger, K.; Böck, F.; Brenner, H. Meningeal Melanocytoma. Report of a Case and Review of the Literature. Acta Neurochir. 1988, 94 . https://doi.org/10.1007/BF01406621.
82. Seo, J.S.; Ahn, S.S.; Choi, J.H.; Choi, H.J. Primary Meningeal Melanocytoma in the Thoracic Spine—A Case Report . Korean J. Spine **2011**, *8*, 121–124.
83. Das, K.; Nair, A.; Jaiswal, S.; Sahu, R.; Srivastava, A.; Kumar, R.; Mehrotra, A. Supratentorial Intermediate Grade Meningeal Melanocytoma with Intratumoral Bleed in the Background of Neurocutaneous Melanosis: Report of an Unusual Case and Review of Literature. Asian J. Neurosurg. **2017**, *12*, 98. https://doi.org/10.4103/1793-5482.145113.
84. Kang, S.-G.; Yoo, D.S.; Cho, K.S.; Kim, D.S.; Chang, E.D.; Huh, P.W.; Kim, M.C. Coexisting Intracranial Meningeal Melanocytoma, Dermoid Tumor, and Dandy-Walker Cyst in a Patient with Neurocutaneous Melanosis. Case Report. J. Neurosurg. **2006**, *104*, 444–447. https://doi.org/10.3171/jns.2006.104.3.444.
85. Khadilkar, U.N.; Agarwal, N.; Deeshma; Bhat, V. Primary Meningeal Melanocytoma. Kathmandu Univ. Med. J. (KUMJ) 6.
86. Kim, O.H.; Kim, S.J.; Choo, H.J.; Lee, S.J.; Lee, I.S.; Kim, J.Y.; Kim, H. Spinal Meningeal Melanocytoma with Benign Histology Showing Leptomeningeal Spread: Case Report. Korean J. Radiol. **2013**, *14*, 470–476. https://doi.org/10.3348/kjr.2013.14.3.470.
87. Kini, J.R.; Jeyraj, V.; Jayaprakash, C.S.; Indira, S.; Naik, C.N.R.; Rao, D. Intraoperative Smear Cytology of Meningeal Melanocytoma of the Posterior Fossa. Cytopathology **2009**, *20*, 59–62. https://doi.org/10.1111/j.1365-2303.2007.00536.x.
88. Kinnen, F.; Fleck, S.K.; Baldauf, J.; Hans, V.; Daeschlein, G.; Rathmann, E.; Schroeder, H.W.S.; Marx, S. Primary Lep-tomeningeal Melanocytic Tumors of the Spine: Report of Two Cases and Review of the Literature. World Neurosurg. **2019**, *124*, 228–236. https://doi.org/10.1016/j.wneu.2019.01.015.
89. Koch, H.J.; Roeber, S.; Zimmermann, U.W.; Schäfer, C.; Villarrubia, V.; Kuchelmeister, K.; Schachenmayr, W.; Bogdahn, U.; Steinbrecher, A. Spinale Und Zerebrale Meningeosis Eines Sekundär Malignisierten Melanozytoms Des Kleinhirnbrückenwinkels. *Wien. Med. Wochenschr.* **2005**, *155*, 360–364.
90. Koenigsmann, M.; Jautzke, G.; Unger, M.; Théallier-Janko, A.; Wiegel, T.; Stoltenburg-Didinger, G. June 2002: 57-Year-Old Male with Leptomeningeal and Liver Tumors Clinical History, Radiology and Microscopic Description. Brain Pathol. **2002**, *12*, 519.
91. Kordás, M.; Czirják, S.; Slowik, F. Primary Meningeal Melanocytoma of the Spinal Cord: Report of a Paediatric Case with Benign Course and Review of the Literature. Eur. J. Neurol. **1996**, *3*, 141–145. https://doi.org/10.1111/j.1468-1331.1996.tb00206.x.
92. Kraft Roverea, R.; Dagnonia, C.; Gomes de Oliveiraa, G.; Sapellia, J. Meningeal Melanocytoma: Case Report and Literature Review. Bol. De La Asoc. Med. De Puerto Rico **2014**, *106*, 30–32.
93. Kuo, K.L.; Lin, C.L.; Wu, C.H.; Chang, C.H.; Tsai, H.P.; Loh, J.K.; Lieu, A.S.; Su, Y.F. Meningeal Melanocytoma Associated with Nevus of Ota: Analysis of Twelve Reported Cases. World Neurosurg. **2019**, *127*, e311–e320. https://doi.org/10.1016/j.wneu.2019.03.113.
94. Kurita, H.; Segawa, H.; Shin, M.; Ueki, K.; Ichi, S.; Sasaki, T.; Tago, M.; Kirino, T. Radiosurgery of Meningeal Melanocytoma. *J. Neuro-Oncol.* **2000**, *46*, 57–61.
95. Küsters-Vandevelde, H.V.N.; Kruse, V.; van Maerken, T.; Boterberg, T.; Pfundt, R.; Creytens, D.; van den Broecke, C.; Machielsen, T.C.; Koelsche, C.; von Deimling, A.; et al. Copy Number Variation Analysis and Methylome Profiling of a GNAQ-Mutant Primary Meningeal Melanocytic Tumor and Its Liver Metastasis. *Exp. Mol. Pathol.* **2017**, *102*, 25–31. https://doi.org/10.1016/j.yexmp.2016.12.006.
96. Lach, B.; Russell, N.; Benoit, B.; Atack, D. Cellular Blue Nevus ("melanocytoma") of the Spinal Meninges: Electron Microscopic and Immunohistochemical Features. Neurosurgery **1988**, *22*, 773–780. https://doi.org/10.1227/00006123-198804000-00030.
97. Leonardi1, M.A.; Lumenta1, C.B.; Èlzle2 , A.S.; Mu, J.; Ècker3 , È.-H. Unusual Clinical Presentation of a Meningeal Melanocytoma with Seizures: Case Report and Review of the Literature. Acta Neurochir. **1998**, *140*, 621–628.

98. Limas, C.; Tio, F.O. Meningeal Melanocytoma ("melanotic Meningioma"). Its Melanocytic Origin as Revealed by Electron Microscopy. *Cancer* **1972**, *30*, 1286–1294. https://doi.org/10.1002/1097-0142(197211)30:5<1286::aid-cncr2820300522>3.0.co;2-v.
99. Lin, B.; Yang, H.; Qu, L.; Li, Y.; Yu, J. Primary Meningeal Melanocytoma of the Anterior Cranial Fossa: A Case Report and Review of the Literature. World J. Surg. Oncol. **2012**, *10*, 135.
100. Lee, S.-U.; Kim, H.-J.; Choi, J.-Y.; Kim, J.-S. Lower Brainstem Melanocytoma Masquerading as Vestibular Paroxysmia. J. Neurol. **2018**, *265*, 1222–1225. https://doi.org/10.1007/s00415-018-8868-x.
101. Litofsky, N.S.; Zee, C.S.; Breeze, R.E.; Chandrasoma, P.T. Meningeal Melanocytoma: Diagnostic Criteria for a Rare Lesion. Neurosurgery **1992**, *31*, 945–950. https://doi.org/10.1227/00006123-199211000-00019.
102. Maaloul, I.; Moussaoui, M.; Salah, A.; Feki, W.; Fourati, H.; Charfi, N.; Mnif, Z. Suprasellar Melanocytoma with Lep-tomeningeal Seeding: An Aggressive Clinical Course for a Histologically Benign Tumor. Case Rep. Radiol. **2021**, *2021*, 7306432. https://doi.org/10.1155/2021/7306432.
103. Maiuri, F.; Iaconetta, G.; Benvenuti, D.; Lamaida, E.; de Caro, M.L. Intracranial Meningeal Melanocytoma: Case Report. Surg. Neurol. **1995**, *44*, 504–509. https://doi.org/10.1016/0090-3019(95)00182-4.
104. Malik, A.; Goyal, M.; Gambhir, M.; Patir, R.; Mishra, N.K.; Gaikwad, S.; Sharma, M.C. Imaging Appearances of Meningeal Melanocytoma. *J. Clin. Neurosci.* **1999**, *6*, 264–265. https://doi.org/10.1016/s0967-5868(99)90520-9.
105. Mangels, K.J.; Johnson, M.D.; Weil, R.J. 35-Year-Old Woman with Progressive Bilateral Leg Weakness. Brain Pathol. **2006**, *16*, 183–184. https://doi.org/10.1111/j.1750-3639.2006.00003_2.x.
106. Marwaha, N.; Batanian, J.R.; Coppens, J.R.; Pierson, M.J.; Richards-Yutz, J.; Ebrahimzadeh, J.; Ganguly, A.; Guzman, M.A. Subcutaneous Melanocytoma Mimicking a Lipoma: A Rare Presentation of a Rare Neoplasm with Histological, Immunohistochemical, Cytogenetic and Molecular Characterization. *J. Cutan. Pathol.* **2016**, *43*, 1186–1196. https://doi.org/10.1111/cup.12808.
107. Mathai, A.M.; Naik, R.; Pai, M.R.; Kini, J.R.; Kumar, S.; Ballal, C.K. Orbital Melanocytoma. Orbit **2008**, *27*, 383–387. https://doi.org/10.1080/01676830802333626.
108. Matsumoto, S.; Kang, Y.; Sato, S.; Kawakami, Y.; Oda, Y.; Araki, M.; Kawamura, J.; Uchida, H. Spinal Meningeal Melanocytoma Presenting with Superficial Siderosis of the Central Nervous System. Case Report and Review of the Literature. J. Neurosurg. **1998**, *88*, 890–894. https://doi.org/10.3171/jns.1998.88.5.0890.
109. Matsuno, H.; Takasu, S.; Seki, Y. Usefulness of Neuromelanin Sensitive MRI for En Plaque Meningeal Melanocytoma Involving the Cavernous Sinus: A Case Report. NMC Case Rep. J. **2019**, *6*, 43–46. https://doi.org/10.2176/nmccrj.cr.2018-0211.
110. Merciadri, P.; Secci, F.; Sbaffi, P.F.; Zona, G. Multifocal Meningeal Melanocytoma of the Conus Medullaris. Acta Neurochir. **2011**, *153*, 2283–2285. https://doi.org/10.1007/s00701-011-1143-x.
111. Mitchell, P.J.; Funt, S.A. Hemiballismus and Meningioma/Pineal and Meningeal Melanomatosis Case Reports.
112. Miura, I.; Kubota, M.; Momosaki, O.; Nyui, M.; Takebayashi, K.; Kawamata, T.; Yuzurihara, M. A Rapidly Growing Cervical Meningeal Melanocytoma with a Dumbbell-Shaped Extension. World Neurosurg. **2020**, *134*, 90–93. https://doi.org/10.1016/j.wneu.2019.10.150.
113. Muñoz-Hidalgo, L.; Lopez-Gines, C.; Navarro, L.; Callaghan, R.C.; San Miguel, T.; Gil-Benso, R.; Quilis, V.; Botella, L.; Gonzalez-Darder, J.; Cerda-Nicolas, M. BRAF V600E Mutation in Two Distinct Meningeal Melanocytomas Associated With a Nevus of Ota. J. Clin. Oncol. Off. J. Am. Soc. Clin. Oncol. **2014**, *32*, e72–e75.
114. Muthappan, M.; Muthu, T.; Hussain, Z.; Lamont, D.; Balakrishnan, V. Cervical Intramedullary Melanocytoma: A Case Report and Review of Literature. J. Clin. Neurosci. **2012**, *19*, 1450–1453. https://doi.org/10.1016/j.jocn.2011.09.040.
115. Lee, N.K.; Lee, J.Y.; Wang, K.C.; Kim, D.G.; Park, S.H.; Cheon, J.E.; Phi, J.H.; Kim, S.K. Primary Atypical Melanocytoma Arising from the Cavernous Sinus in a Child. *Child's Nerv. Syst.* **2015**, *31*, 1577–1582. https://doi.org/10.1007/s00381-015-2741-3.
116. Nakahara, K.; Morota, N.; Ihara, S.; Oka, H.; Matsuoka, K.; Nakagawa, A. Meningeal Melanocytoma Extruded From the Skull of a Neonate-Case Report. Neurol. Med. -Chir. **2010**, *50*, 240–242.
117. Navas, M.; Pascual, J.M.; Fraga, J.; Pedrosa, M.; Shakur, S.; Carrasco, R.; Martínez, P.; Manzanares, R.; Sola, R.G. Intracranial Intermediate-Grade Meningeal Melanocytoma with Increased Cellular Proliferative Index: An Illustrative Case Associated with a Nevus of Ota. *J. Neurooncol.* **2009**, *95*, 105–115. https://doi.org/10.1007/s11060-009-9907-3.
118. Nicolotto, E.; Presas-Rodriguez, S.; Morra, I.; Franchino, F.; Magistrello, M.; Pellerino, A.; Massaro, F.; Pinessi, L.; Rudà, R.; Soffietti, R. Spinal Melanocytoma with Leptomeningeal Spread and Long Survival Following Surgery and Chemotherapy. J. Neurosurg. Sci. **2018**, *62*, 375–378. https://doi.org/10.23736/S0390-5616.16.03687-0.
119. Offiah, C.J.; Laitt, R.D. Intracranial Meningeal Melanocytoma: A Cause of High. Signal on T1-and Low Signal on T2-Weighted MRI. Clin. Radiol. **2006**, *61*, 294–298.
120. Oruckaptan, H.H.; Soylemezoglu, F.; Kutluk, T.; Akalan, N. Benign Melanocytic Tumor in Infancy: Discussion on a Rare Case and Review of the Literature. *Pediatr. Neurosurg.* **2000**, *32*, 240–247. https://doi.org/10.1159/000028945.
121. Painter, T.J.; Chaljub, G.; Sethi, R.; Singh, H.; Gelman, B. Intracranial and Intraspinal Meningeal Melanocytosis. *Am. J. Neuroradiol.* **2000**, *21*, 1349–1353.
122. Palicelli, A.; Disanto, M.G.; Panzarasa, G.; Veggiani, C.; Galizia, G.; Dal Cin, S.; Gruppioni, E.; Boldorini, R. Orbital Meningeal Melanocytoma: Histological, Immunohistochemical and Molecular Characterization of a Case and Review of the Literature. *Pathol. Res. Pract.* **2016**, *212*, 946–953. https://doi.org/10.1016/j.prp.2016.07.009.
123. Pan, H.; Wang, H.; Fan, Y. Intracranial Meningeal Melanocytoma Associated with Nevus of Ota. J. Clin. Neurosci. **2011**, *18*, 1548–1550. https://doi.org/10.1016/j.jocn.2011.01.040.

124. Perrini, P.; Caniglia, M.; Pieroni, M.; Castagna, M.; Parenti, G.F. Malignant Transformation of Intramedullary Melanocytoma: Case Report. *Neurosurgery* **2010**, *67*, E867–E869. https://doi.org/10.1227/01.NEU.0000372919.96651.34.
125. Phang, I.; Elashaal, R.; Ironside, J.; Eljamel, S. Primary Cerebellopontine Angle Melanocytoma: Review. *J. Neurol. Surg. Rep.* **2012**, *73*, 025–031. https://doi.org/10.1055/s-0032-1311756.
126. Piercecchi-Marti, M.-D.; Mohamed, H.; Liprandi, A.; Gambarelli, D.; Grisoli, F.; Pellissier, J.-F. Intracranial Meningeal Melanocytoma Associated with Ipsilateral Nevus of Ota. Case Report. J. Neurosurg. **2002**, *96*, 619–623. https://doi.org/10.3171/jns.2002.96.3.0619.
127. Pierson, M.; Marwaha, N.; Guzman, M.; Mikulec, A.A.; Coppens, J.R. Multifocal Melanocytoma of the Posterior Fossa and Subcutaneous Scalp in the Absence of Neurocutaneous Melanosis. *Surg. Neurol. Int.* **2016**, *7*, S591–S595. https://doi.org/10.4103/2152-7806.189728.
128. Prabhu, S.S.; Lynch, P.G.; Keogh, A.J.; Parekh, H.C. Intracranial Meningeal Melanocytoma: A Report of Two Cases and a Review of the Literature. Surg. Neurol. **1993**, *40*, 516–521. https://doi.org/10.1016/0090-3019(93)90058-9.
129. Rades, D.; Heidenreich, F.; Tatagiba, M.; Brandis, A.; Karstens, J.H. Therapeutic Options for Meningeal Melanocytoma. Case Report. *J. Neurosurg.* **2001**, *95*, 225–231. https://doi.org/10.3171/spi.2001.95.2.0225.
130. Rahimi-Movaghar, V.; Karimi, M. Meningeal Melanocytoma of the Brain and Oculodermal Melanocytosis (Nevus of Ota): Case Report and Literature Review. *Surg. Neurol.* **2003**, *59*, 200–210. https://doi.org/10.1016/s0090-3019(02)01052-2.
131. Rai, S.; Sharma, M.; Naik, R.; Sinha, R.; Philipose, R.; Verghese, R. Melanocytoma of Cerebellum. Indian J. Pathol. Microbiol. **2008**, *51*, 47. https://doi.org/10.4103/0377-4929.40394.
132. Reddy, R.; Krishna, V.; Prasad Sahu, B.; Uppin, M.; Sundaram, C. Multifocal Spinal Meningeal Melanocytoma: An Illustrated Case Review. Turk. Neurosurg. **2012**, *22*, 791–794. https://doi.org/10.5137/1019-5149.JTN.4322-11.1.
133. Roser, F.; Nakamura, M.; Brandis, A.; Hans, V.; Vorkapic, P.; Samii, M. Transition from Meningeal Melanocytoma to Primary Cerebral Melanoma. Case Report. *J. Neurosurg.* **2004**, *101*, 528–531. https://doi.org/10.3171/jns.2004.101.3.0528.
134. Ren, Y.; Xiao, A.; Wu, X.; Zhang, Y. Meningeal Melanocytoma of the Middle Cranial Fossa (the Meckel's Cave). Neurol. India **2015**, *63*, 260.
135. Rousseau, A.; Bernier, M.; Kujas, M.; Varlet, P. Primary Intracranial Melanocytic Tumor Simulating Pituitary Macroadenoma: Case Report and Review of the Literature. Neurosurgery **2005**, *57*, E369. https://doi.org/10.1227/01.neu.0000166686.19823.a9.
136. Ruelle, A.; Tunesi, G.; Andrioli, G. Spinal Meningeal Melanocytoma. Case Report and Analysis of Diagnostic Criteria. Neurosurg. Rev. **1996**, *19*, 39–42.
137. Rutten, I.; Bolle, S.; Kaschten, B.; Stevenaert, A.; Deneufbourg, J.M.; Deprez, M. Recurrent Intracranial Melanocytoma Associated with a Nevus of Ota. Acta Neurochir. **2005**, *147*, 313–315. https://doi.org/10.1007/s00701-004-0457-3.
138. Sakar, M.; Erdogan, O.; Bozkurt, S.U.; Dagcinar, A. Intermediate-Grade Meningeal Melanocytoma in a 19 Months Old Child with Difficulties in Differential Diagnosis and Management. *Turk. Neurosurg.* **2022**, 32, 166-170. https://doi.org/10.5137/1019-5149.jtn.34672-21.4.
139. Sakata, K.; Miyoshi, J.; Takeshige, N.; Komaki, S.; Miyagi, N.; Nakashima, S.; Morioka, M.; Sugita, Y. Primary Meningeal Melanocytoma of the Sellar Region: Review of the Literature and Differential Diagnosis with Special Reference to Angio-graphical Features. Pituitary **2015**, *18*, 685–694. https://doi.org/10.1007/s11102-015-0635-8.
140. Salah El-Din, A.M.; Aboul-Ela, H.M.; Alsawy, M.F.; Koheil, A.; Ashry, A.H. Spinal Meningeal Melanocytoma in a 5-Year-Old Child: A Case Report and Review of Literature. Egypt. J. Neurol. Psychiatry Neurosurg. **2018**, *54*, 13. https://doi.org/10.1186/s41983-018-0017-z.
141. Samadian, M.; Nejad, A.M.; Bakhtevari, M.H.; Sabeti, S.; Sharifi, G.; Jabbari, R.; Rezaei, O. Primary Meningeal Melanocytoma in the Left Temporal Lobe Associated with Nevus Ota: A Case Report and Review of the Literature. World Neurosurg. **2015**, *84*, 567–573. https://doi.org/10.1016/j.wneu.2015.03.061.
142. Samimi, K.; Gharib, M.H.; Rezaei-Kalantari, K.; Jafari, M. Unusual Tonsillar Herniation in Meningeal Melanocytoma: A Case Report. Iran. J. Radiol. **2012**, *9*, 227–230. https://doi.org/10.5812/iranjradiol.8766.
143. San-Miguel, T.; Navarro, L.; Sanchez-Sendra, B.; Megıas, J.; Muñoz-Hidalgo, L.; Santonja, N.; Lopez-Gines, C.; Cerda-Nicolas, M. Identification of a Novel BRCA1 Alteration in Recurrent Melanocytoma Resulting in Increased Proliferation. *J. Neuropathol. Exp. Neurol.* **2020**, *79*, 1233–1238. https://doi.org/10.1093/JNEN/NLAA089.
144. Sankhla, S.K.; Lynch, P.G.; Davis, C.H. Spinal Meningeal Melanocytoma: A Case Report and Review of the Literature. Br. J. Neurosurg. **1996**, *10*, 205–210. https://doi.org/10.1080/02688699650040395.
145. Sato, K.; Kubota, T.; Kodera, T.; Kitai, R.; Takeuchi, H.; Yoshida, K. Melanocytoma in the Orbital Apex. *J. Neurooncol.* **2009**, *92*, 107–110. https://doi.org/10.1007/s11060-008-9723-1.
146. Schembri, M.; Kok, H.K.; Brennan, P.; O'Hare, A.; Thornton, J.; Looby, S.; Asadi, H. Mystery Case: Dural Melanocytoma with Leptomeningeal Melanocytosis. Neurology **2017**, *88*, e70–e71.
147. Schindler, C.U.; Kuchelmeister, K.; Richter, H.P.; Schachenmayr, W. Meningeal Melanocytoma. Pathologe 1998, 19 . https://doi.org/10.1007/s002920050292.
148. Sethi, D.; Duhan, A.; Sen, R.; Goyal, V.; Modi, S. Spinal Meningeal Melanocytoma. Asian J. Neurosurg. **2011**, *6*, 111. https://doi.org/10.4103/1793-5482.92176.
149. Shaikh, S.; Gupta, G.; Mohanty, C.; Deopujari, C. Spinal Nerve Root Extradural Melanocytoma Progressing to Malignant Melanoma: A Case Report with Review of Literature. *Asian J. Neurosurg.* **2021**, *16*, 394. https://doi.org/10.4103/ajns.ajns_416_20.

150. Shanthi, V.; Ramakrishna, B.A.; Bheemaraju, V.V.; Rao, N.M.; Murthy Athota, V.R. Spinal Meningeal Melanocytoma: A Rare Meningeal Tumor. Ann. Indian Acad. Neurol. **2010**, *13*, 308–310. https://doi.org/10.4103/0972-2327.74192.
151. Sharma, V.; Bhaskar, S.; Kumar, A.; Bhardwaj, M. Conus Melanocytoma: A Rare Spinal Tumor. Neurology **2019**, *67*, 591.
152. Shimoda, H.; Oka, K.; Naoi, Y.; Nishida, S.; Oka, T.; Nakazato, Y.; Mori, N. Primary Melanocytoma Arising from the Thoracic Leptomeninges Case. Clin. Neuropathol. **1999**, *18*, 80–83.
153. Shin, D.; Lee, K.J.; Adeluwa, T.; Hur, J. Machine Learning-Based Predictive Modeling of Postpartum Depression. J. Clin. Med. **2020**, *9*, 2899. https://doi.org/10.3390/jcm9092899.
154. Shinoda, K.; Hayasaka, S.; Nagaki, Y.; Kadoi, C.; Kurimoto, M.; Okada, E. Melanocytoma of the Left Optic Nerve Head and Right Retrobulbar Optic Neuropathy Compressed by a Tuberculum Sellae Meningioma. Ophthalmologica **2000**, *214*, 161–163. https://doi.org/10.1159/000027489.
155. Shownkeen, H.N.; Harmath, C.; Thomas, C. Multiform Cervical Melanocytoma: A Case Report. Neuroradiology **2002**, *44*, 1008–1010. https://doi.org/10.1007/s00234-002-0875-y.
156. Srirama Jayamma, S.; Sud, S.; Buxi, T.; Madan, V.; Goyal, A.; Dhawan, S. Cervical Spinal Meningeal Melanocytoma Presenting as Intracranial Superficial Siderosis. Case Rep. Radiol. **2015**, *2015*, 674868. https://doi.org/10.1155/2015/674868.
157. Steinberg, J.M.; Gillespie, J.J.; MacKay, B.; Benjamin, R.S.; Leavens, M.E. Meningeal Melanocytoma with Invasion of the Thoracic Spinal Cord. Case Report. *J. Neurosurg.* **1978**, *48*, 818–824. https://doi.org/10.3171/jns.1978.48.5.0818.
158. Sun, J.; Wang, C.; Shu, Q.; Liu, J.; Mao, G. Case Report A Case Report on Intermediate-Grade Malignant Meningeal Melanocytoma with Elevated Cell Proliferation Index. Int. J. Clin. Exp. Pathol. **2015**, *8*, 11698.
159. Sung Hye Park, M.D.; Heum Rye Park, M.D.; Yong Ko, M.D. Spinal Meningeal Melanocytoma. J. Korean Med. Sci. **1992**, *7*, 364–368.
160. O'Brien, T.F.; Moran, M.; Miller, J.H.; Hensley, S.D. Meningeal Melanocytoma. An Uncommon Diagnostic Pitfall in Surgical Neuropathology. Arch. Pathol. Lab. Med. **1995**, *119*, 542–546.
161. Tatagiba, M.; Böker, D.K.; Brandis, A.; Samii, M.; Ostertag, H.; Babu, R. Meningeal Melanocytoma of the C8 Nerve Root: Case Report. Neurosurgery **1992**, *31*, 958–961. https://doi.org/10.1227/00006123-199211000-00023.
162. Tateyama, M.; Fujimoto, T.; Nakamura, T.; Miyamoto, T. Meningeal Melanocytoma Occurring at Epidural Region of the Cervical Spine. Spine Surg. Relat. Res. **2020**, *4*, 377–379. https://doi.org/10.22603/SSRR.2020-0027.
163. Tewari, M.K.; Radotra, B.D.; Sharma, B.S.; Mathuriya, S.N.; Pathak, A.; Banerjee, A.K.; Kak, V.K. Meningeal Melanocytoma: Report of Two Cases. Indian J. Cancer 1990, 27 .
164. Tregnago, A.C.; Furlan, M.v.; Bezerra, S.M.; Porto, G.C.L.M.; Mendes, G.G.; Henklain, J.V.R.; Pinto, C.A.L.; Kowalski, L.P.; de Carvalho, G.B.; Costa, F.D. Orbital Melanocytoma Completely Resected with Conservative Surgery in Association with Ipsilateral Nevus of Ota: Report of a Case and Review of the Literature. Head Neck **2015**, *37*, E49–E55. https://doi.org/10.1002/hed.23828.
165. Tsai, M.-H.; Lin, W.-P.; Liao, W.-A.; Chiang, P.-Y.; Lin, Y.-C. Recurrent Spinal Meningeal Melanocytoma at Lumbar Spine Level: A Case Report. Br. J. Neurosurg. 2021 . https://doi.org/10.1080/02688697.2020.1867062.
166. Tsugu, H.; Nabeshima, K.; Matsumoto, S.; Omura, T.; Yahiro, T.; Oshiro, S.; Komatsu, F.; Abe, H.; Fukushima, T.; Inoue, T.; et al. A Case of a Heavily Pigmented Orbital Melanocytoma. Brain Tumor Pathol. **2009**, *26*, 25–29. https://doi.org/10.1007/s10014-008-0242-8.
167. Turhan, T.; Oner, K.; Yurtseven, T.; Akalin, T.; Ovul, I. Spinal Meningeal Melanocytoma. Report of Two Cases and Review of the Literature. J. Neurosurg. **2004**, *100*, 287–290.
168. Uematsu, Y.; Yukawa, S.; Yokote, H.; Itakura, T.; Hayashi, S.; Komai, N. Meningeal Melanocytoma: Magnetic Resonance Imaging Characteristics and Pathological Features. Case Report. J. Neurosurg. **1992**, *76*, 705-710. https://doi.org/10.3171/jns.1992.76.4.0705.
169. Uozumi, Y.; Kawano, T.; Kawaguchi, T.; Kaneko, Y.; Ooasa, T.; Ogasawara, S.; Yoshida, H.; Yoshida, T. Malignant Transformation of Meningeal Melanocytoma: A Case Report. *Brain Tumor Pathol.* **2003**, *20*, 21–25.
170. URAMARU, K.; SAKATA, K.; SHIMOHIGOSHI, W.; KAWASAKI, T.; MANAKA, H. Primary Meningeal Melanocytoma Located in the Craniovertebral Junction: A Case Report and Literature Review. NMC Case Rep. J. **2021**, *8*, 349–354. https://doi.org/10.2176/nmccrj.cr.2020-0191.
171. Vaidya, M.; Dhake, R.; Parikh, R.; Sabnis, M.; Sabnis, J. Recurrent Meningeal Melanocytoma of Cervical Spine: A Rare Case. Asian J. Neurosurg. **2021**, *16*, 159. https://doi.org/10.4103/ajns.ajns_327_20.
172. Verma, D.S.; Spitzer, G.; Legha, S.; McCredie, K.B. Chemoimmunotherapy for Meningeal Melanocytoma of the Thoracic Spinal Cord. Report of a Case. *JAMA* **1979**, *242*, 2435–2436.
173. Vreto, G.; Rroji, A.; Xhumari, A.; Leka, L.; Rakacolli, M.; Petrela, M. Meningeal Melanocytoma of the Cerebellopontine Angle as the Unusual Cause of Superficial Siderosis. Neuroradiology **2011**, *53*, 927–930.
174. Winston, K.R.; Sotrel, A.; Schnitt, S.J. Meningeal Melanocytoma. Case Report and Review of the Clinical and Histological Features. J. Neurosurg. **1987**, *66*, 50–57. https://doi.org/10.3171/jns.1987.66.1.0050.
175. Xie, S.; Jiang, Z. Primary Spinal Cord Melanocytoma: A Case Report and Review of Literature. Int. J. Clin. Exp. Pathol. **2019**, *12*, 669–673.

176. Yang, C.; Fang, J.; Li, G.; Jia, W.; Liu, H.; Qi, W.; Xu, Y. Spinal Meningeal Melanocytomas: Clinical Manifestations, Radiological and Pathological Characteristics, and Surgical Outcomes. J. Neurooncol. **2016**, *127*, 279–286. https://doi.org/10.1007/s11060-015-2006-8.
177. Yin, M.; Ma, J.; Ye, J.; Xu, H.; Mo, W. 8-Year Follow-up for Woman with Spinal Meningeal Melanocytoma in S1 Nerve Root: Case Report and Literature Review. World Neurosurg. **2019**, *129*, 143–147. https://doi.org/10.1016/j.wneu.2019.05.147.
178. Yukawa, H.; Seki, H.; Sugawara, T.; Boku, N.; Higuchi, H.; Ono, S. A Case of Primary Meningeal Melanocytoma of the Left Middle Fossa. No Shinkei Geka **2003**, *31*, 1023–1028.
179. Zeiler, F.A.; Krcek, J.P. Plaque-Type Blue Nevus with Meningeal Melanocytomas. Can. J. Neurol. Sci. **2012**, *39*, 117–120.
180. Liu, Z.-Q. World Journal of Clinical Cases. Contents Thrice Mon. **2021**, *9*, 8280–8626.
181. Zhou, H.W.; Tran, A.Q.; North, V.S.; Zagzag, D.; Sen, C.; Kazim, M. GNA11 Mutation in an Intracranial Melanocytoma with Orbital Involvement and Nevus of Ota. *Ophthalmic. Plast. Reconstr. Surg.* **2021**, *38*, e47–e49. https://doi.org/10.1097/IOP.0000000000002081.
